# Integrated multi-omics analysis of Alzheimer’s disease shows molecular signatures associated with disease progression and potential therapeutic targets

**DOI:** 10.1038/s41598-023-30892-6

**Published:** 2023-03-06

**Authors:** Pradeep Kodam, R. Sai Swaroop, Sai Sanwid Pradhan, Venketesh Sivaramakrishnan, Ramakrishna Vadrevu

**Affiliations:** 1 Department of Biological Sciences, Birla Institute of Technology and Science Pilani, Hyderabad Campus, Jawahar Nagar, Hyderabad, Telangana 500078 India; 2 Disease Biology Lab, Department of Biosciences, Sri Sathya Sai Institute of Higher Learning, Prasanthi Nilayam, Anantapur, Andhra Pradesh 515134 India

**Keywords:** Biochemical reaction networks, Cellular signalling networks, Data integration, Data mining, Gene ontology, Gene regulatory networks, Literature mining, Microarrays, Neurodegenerative diseases

## Abstract

Alzheimer’s disease (AD) is a progressive neurodegenerative disease characterized by the formation of amyloid plaques implicated in neuronal death. Genetics, age, and sex are the risk factors attributed to AD. Though omics studies have helped to identify pathways associated with AD, an integrated systems analysis with the available data could help to understand mechanisms, potential biomarkers, and therapeutic targets. Analysis of transcriptomic data sets from the GEO database, and proteomic and metabolomic data sets from literature was performed to identify deregulated pathways and commonality analysis identified overlapping pathways among the data sets. The deregulated pathways included those of neurotransmitter synapses, oxidative stress, inflammation, vitamins, complement, and coagulation pathways. Cell type analysis of GEO data sets showed microglia, endothelial, myeloid, and lymphoid cells are affected. Microglia are associated with inflammation and pruning of synapses with implications for memory and cognition. Analysis of the protein-cofactor network of B_2_, B_6,_ and pantothenate shows metabolic pathways modulated by these vitamins which overlap with the deregulated pathways from the multi-omics analysis. Overall, the integrated analysis identified the molecular signature associated with AD. Treatment with anti-oxidants, B_2_, B_6_, and pantothenate in genetically susceptible individuals in the pre-symptomatic stage might help in better management of the disease.

## Introduction

Alzheimer’s disease (AD) is a progressive neurological disorder characterized by the formation of amyloid plaques, tau tangles, and synaptic and neuronal dysfunction^1^. The major risk factors of AD include age, sex, and genetic variants^2^. Rare coding variants in APP(Amyloid precursor protein), PSEN1 (Presenilin 1), and PSEN2 (Presenilin 2) increase the risk for late-onset of AD^3^. Genetic variants of SLC9C1, CSN1S1, and LOXL4 though recessive, delay the age of onset of AD^4^. The ε4 allele of apolipoprotein E (ApoE), involved in cholesterol metabolism, which is secreted by astrocytes is strongly associated with the late onset of AD^5^. ApoE isoforms have a role in controlling brain glucose and lipid metabolism, neuronal signaling, inflammation, and mitochondrial function and regulate Aβ aggregation and clearance^6,7^. ApoE4 is associated with gene expression changes in all cell types of the human brain and aberrant deposition of cholesterol in oligodendrocytes leads to reduced myelination^8^. ApoE-expressing microglia promote neurodegeneration by disrupting the phagocytosis of Aβ aggregates^9^. Mutations in APP, PSEN1 & 2, ADAM10, and ADAM1J cause the formation of Aβ plaques and are sufficient to cause the whole biochemical and morphological characteristic of AD^2^. However, studies have also shown that the toxicity is caused due to soluble monomers or oligomers^10^. AD is associated with changes in brain volume, and atrophy in the hippocampus and entorhinal cortex at the early stages of disease progression^11^. The disease manifestations include motor, behavioral, cognitive, and memory dysfunction^5^. Current medications are restricted to the management of symptoms and disease-modifying therapies with antibodies such as aducanumab and lecanumab is shown to reduce the amyloid plaques in AD patients which influence the course of disease^12,13^. Hence understanding the factors that contribute to amyloid formation will help to achieve mechanistic insights and also help to identify potential biomarkers and therapeutic targets which will aid in the better management of the disease^14^. Studies have shown impaired energy metabolism in AD which could act both as biomarkers and therapeutic target in disease^15^. Previous studies have also shown that brain volume and metabolic changes precede the onset of symptoms in neurodegenerative diseases like AD and Huntington’s disease (HD)^16,17^.

Mitochondrial impairment, cellular energy deficiencies, and oxidative damage are found to be important in the pathogenesis of AD^18,19^. Studies have reported the presence of Aβ in the cells and mitochondria. In mitochondria, Aβ binds to the heme group leading to the production of free radicals, thus causing oxidative damage and cell death^20^. Bioenergetic deficits and oxidative stress are the key contributors to cognitive decline in AD^21^. The mitochondrial enzymes (cytochrome c oxidase, COX) that are important for energy metabolism is deficient in AD brains^22^.

Consistent with mitochondrial dysfunction, AD is associated with metabolic remodeling^23^. Glycolysis was found to be upregulated in AD^24^. Levels of lactate and lactate transporters are altered in AD mouse models resulting in deregulated astrocyte-neuron lactate shuttle^25^. AD brain was shown to exhibit hypometabolism decades before the manifestation of symptoms suggesting the role of metabolic dysfunction in AD^26^. Fluorodeoxyglucose-positron emission tomography (FDG-PET) studies have shown a reduction in glucose metabolism at the mild cognitive impairment (MCI) stage of AD progression^27^. Magnetic resonance imaging studies have shown decreased levels of glutamate in the AD brain which is crucial for the functioning of the central nervous system (CNS). The astrocyte-neuron glutamine-glutamate shuttle was also found to be affected in AD^28^. A decrease in metabolite levels of N-acetyl aspartate/Creatinine (NAA/Cr) ratio is also observed in AD^29^. The data from the literature are suggestive of metabolic pathways as potential modifiers of AD. Consistent with metabolic deregulation, changes in the levels of various vitamins like B_2_, B_5_, B_6_, B_12_ and B_9_ are reported in AD^30–33^. The vitamins act as cofactors in various enzymatic reactions and low levels of these vitamins are associated with many diseases including neurodegenerative diseases^34^. For many genetic diseases with non-synonymous single nucleotide polymorphisms (SNPs) in the genes encoding enzymes, vitamin/cofactor supplementation is used as the standard of care treatment^35^. Previous studies have shown that supplementation of B_2_, B_5_ and B_6_ was shown to improve symptoms in AD model system^36–38^. The vitamins might potentially act by helping to achieve metabolic homeostasis though this remains to be answered^39^. Previous studies have shown that metabolic deregulation precedes onset of symptoms in AD^40^.

Earlier omics analysis carried out on the samples obtained from AD patients and model systems has shown the associated deregulated pathways with diseases^41^. Transcriptomics profiling of late-onset Alzheimer’s affected brain tissue revealed pathways associated with neural communication, cerebral vasculature, and amyloid-beta clearance are deregulated^42^. Blood transcriptomic analysis has shown an association of AD at different stages with type 2 diabetes^43^. Single-cell transcriptomics analysis revealed myelination-related processes were deregulated in multiple cell types like neurons, astrocytes, oligodendrocytes and microglia^44^. An individual’s metabolome reflects the culmination of the cumulative alterations in genomics, transcriptomic and proteomic profile. In addition, it is also influenced by the environment and lifestyle of an individual^45^. Metabolomic analysis of different kinds of samples of AD patients also indicates deregulation of the Krebs cycle, mitochondrial function, neurotransmitter and amino acid metabolism, and lipid biosynthesis pathways^46–48^. Multi-omic analysis and validation has helped to understand mechanisms, biomarkers and potential therapeutic targets in osteonecrosis, rheumatoid arthritis, glaucoma^49–51^.

Although the multi-omics approach enabled the comprehensive understanding of deregulated pathways associated with the disease. An integrated systems analysis using the available multi-omics data sets can potentially shed light on the interactions of the different molecular processes with further implications for the disease and could also help identify markers, early prognosis, and new therapeutic targets. In the present study an integrative analysis of transcriptomics, proteomics, and metabolomics of blood, brain, and cerebrospinal fluid samples obtained from patient datasets was performed. In addition, proteomics datasets of the brain and blood, and metabolomic datasets of the brain, blood, and cerebrospinal fluid of mice were analysed. The overall analysis from this approach shows the deregulation of inflammation, complement and coagulation, signalling, and metabolic pathways as well as vitamin B_2_, B_6_, and pantothenate pathways. The cell type analysis showed endothelial, microglial, myeloid, and lymphoid cells. Since vitamins act as cofactors, their implications for enzyme activity associated with the deregulation of metabolic pathways have been analysed using a cofactor-protein interaction network using Cytoscape. The results are discussed in light of the implications of deregulated pathways for disease, symptoms and therapeutic targets.

## Results

### Analysis of transcriptomic data sets from the GEO database shows deregulated pathways and cell types with potential implications for disease

The GEO (Gene Expression Omnibus) data sets GSE5281^52^, GSE36980^53^, GSE44770^54^, GSE48350^55^, and GSE140829^56^ from Humans were used for analysis. All the data sets were from the brain except for GSE140829 which is from the peripheral blood data set. The data sets were analysed using Enrichr^57,58^ as given in methods and genes are binned into pathways. To corroborate the gene expression profile to different cell types that are associated with the disease, we carried out cell type analysis using Enrichr as given in the methods. The kinases and transcription factors were predicted using X2K^59^ on all the GEO datasets as described in the methods. The significant differential genes with an adjusted p-value of 0.05 were used for all analyses. Pathway and transcription factor analysis was performed for up-regulated and downregulated genes separately and provided in Supplementary file 12.

### GSE5281

**Figure 1:**
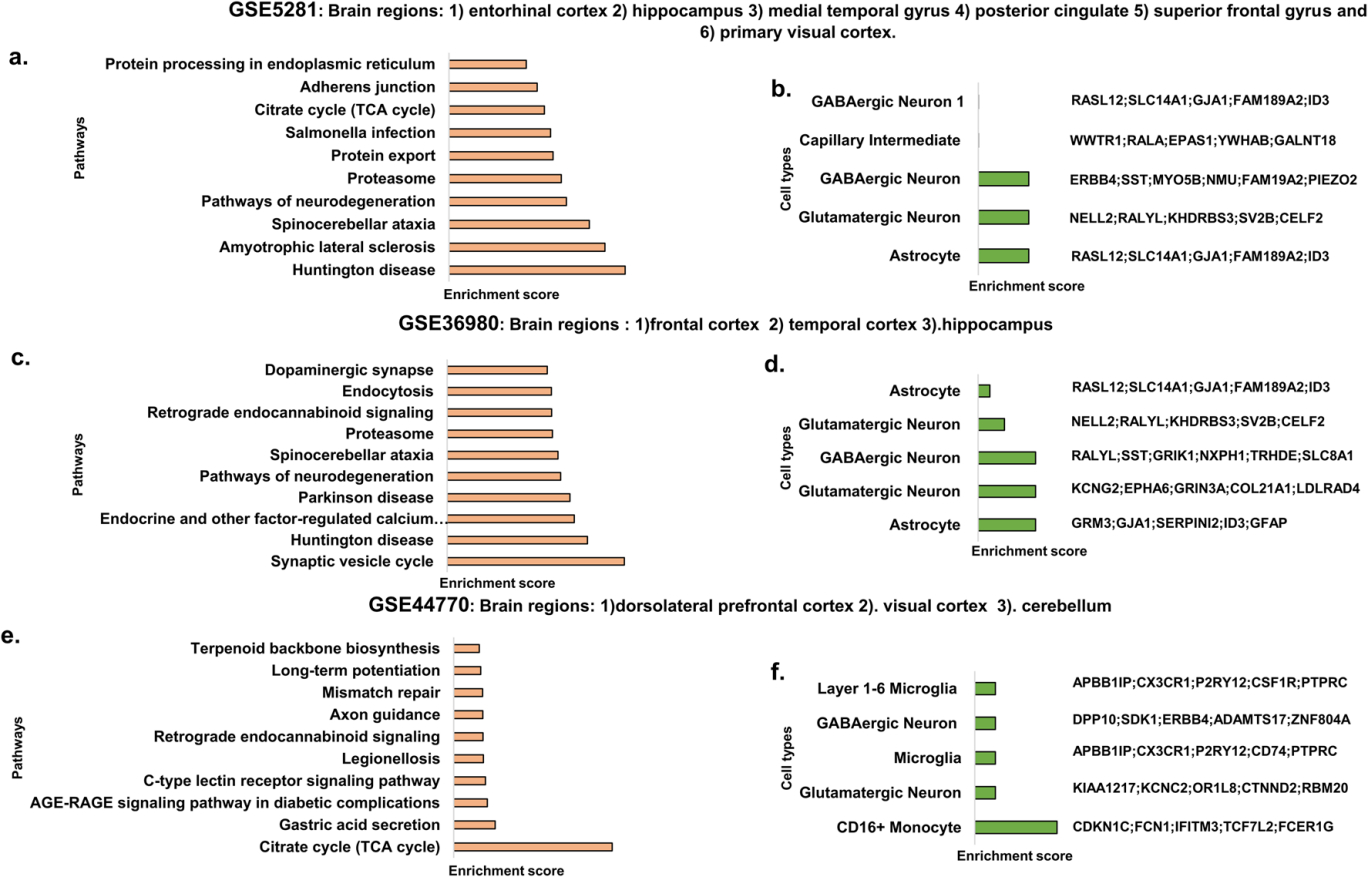
Gene expression data analysis of GSE5281, GSE36980, and GSE44770 GEO datasets. Pathway enrichment analysis of differentially expressed genes by Enrichr tool. Top significant pathways from datasets of (**a**) GSE5281 (**c**). GSE36980 (**e**). GSE44770. Cell type analysis and genes associated with each cell type (**b**). GSE5281 (**d**). GSE36980 (**f**) GSE44770. Figure generated using**,** Microsoft powerpoint 2019 MSO (Version 2212 Build 16.0.15928.20196) 64-bit.

Represent the pooled data sets from the brain regions: Entorhinal cortex, hippocampus, medial temporal gyrus, posterior cingulate, superior frontal gyrus, and Primary visual cortex. Analysis of the differentially expressed significant genes using Enrichr binned them into pathways. The top significant pathways include pathways involved in the protein life cycle, neurodegenerative diseases like spinocerebellar ataxia, amyotrophic lateral sclerosis (ALS), Huntington’s disease (HD), citric acid cycle, and pathways involved in infection (Fig. 1a and Supplementary 1). Pathway Analysis of upregulated genes showed AGE-RAGE and p53 signalling pathways, different viral infection and cancer pathways. The pathways obtained using the down regulated genes involved include various neurodegenerative diseases like ALS, HD, PD, prions, spinocerebellar ataxia, proteasome and oxidative phosphorylation (Supplementary 2) The transcription factors involved include TAF1, BRCA, CREB, MYC, etc. (Supplementary 3 and 5). Further, analysis of kinases involved shows CDKs, MAPKs, ERK, DNAPK ATR/ATM, etc. (Supplementary 4 and 5). The cell types involve astrocytes, glutamatergic and GABAergic neurons, oligodendrocytes and, capillary intermediate neurons (Fig. 1b and Supplementary 6).

### GSE36980

Dataset represents the pooled datasets from brain regions frontal cortex, temporal cortex, and hippocampus. Differentially expressed genes are binned into pathways. The top significant pathways involved Synapse based pathways like Synaptic vesicle cycle, Dopaminergic synapse, neurodegenerative diseases such as (Huntington’s disease, Parkinson’s disease, spinocerebellar ataxia, pathways of neurodegeneration), proteasome, endocytosis, and signaling pathways (Fig. 1c and Supplementary 1). Pathway analysis of upregulated genes showed cytokine and complement and coagulation cascades, bacterial and viral infection and immune pathways. The pathways obtained using the downregulated genes involved include neurodegenerative diseases such as HD, ALS, PD, spinocerebellar ataxia and pathways of neurodegeneration (Supplementary 2). The transcription factors involved include REST, RCOR1, ZNF384, SMAD4, etc. (Supplementary 3 and 5). Further, analysis of kinases showed MAPKs, CDKs, JNK2, GSK3B, HIPK2, etc. (Supplementary 4 and 5). The cell types involve astrocytes, glutamatergic and GABAergic neurons (Fig. 1d and Supplementary 6).

### GSE44770

Dataset represents the pooled datasets from brain regions dorsolateral prefrontal cortex, visual cortex, and cerebellum. Differentially expressed genes are binned into pathways. The top significant pathways involved signaling pathways like retrograde endocannabinoid signalling, AGE-RAGE signaling pathway in diabetic complications, C-type lectin receptor signaling pathway, long-term potentiation, axon guidance, gastric acid secretion, citric acid cycle, pathways involved in infection, and DNA repair (Fig. 1e and Supplementary 1). Pathway analysis of upregulated genes showed viral, bacteria and protozoan infection pathways, AGE-RAGE signalling and osteoclast differentiation. Pathways obtained from downregulated genes include neurodegenerative diseases like PD, HD, pathways of neurodegeneration, nicotine addiction, citrate cycle and retrograde endocannabinoid signalling. (Supplementary 2) The transcription factors involved include SUZ12, AR, GATA1, SOX2, etc. (Supplementary 3 and 5). Further analysis of kinases involved MAPKs, CSNK2A1, GSK3BETA, CDC2, etc. (Supplementary 4 and 5). The cell types involve glutamatergic and GABAergic neurons, monocytes, and microglia (Fig. 1f and Supplementary 6).

### GSE48350

**Figure 2:**
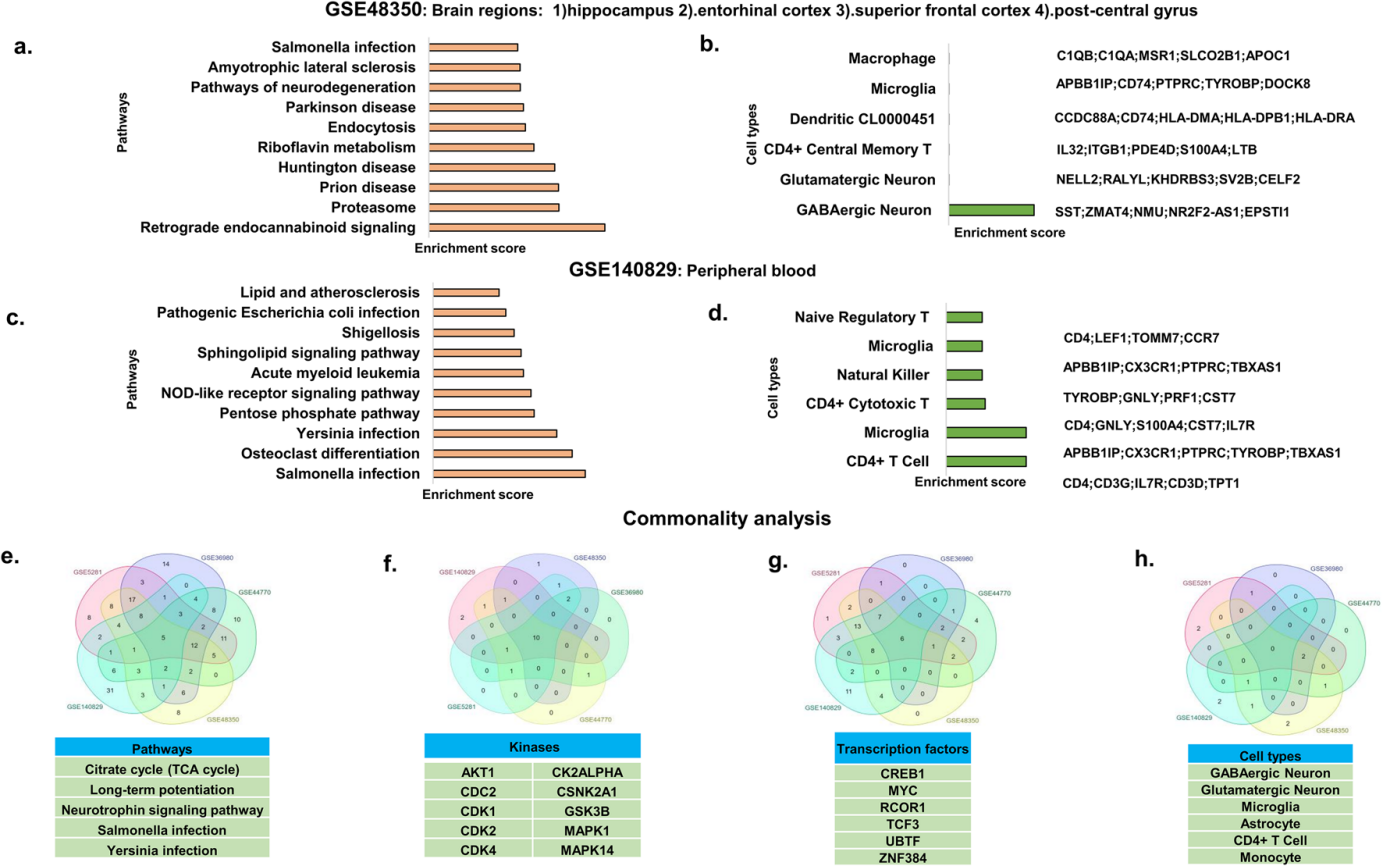
Gene expression data analysis of GSE48350, and GSE140829 GEO datasets. Pathway enrichment analysis of differentially expressed genes by Enrichr tool Top significant pathways from dataset (**a**)**.** GSE48350 (**c**)**.** GSE3140829. Cell type analysis and genes associated with each cell type (**b**). GSE48350 (**d**). GSE140829. Commonality analysis for (**e**)**.** Pathways (**f**). Kinases (**g).** Transcription factors (**h**). Cell types. Figure generated using**,** Microsoft powerpoint 2019 MSO (Version 2212 Build 16.0.15928.20196) 64-bit. Multiple list comparator (https://molbiotools.com/listcompare.php).

Represent the pooled data sets from the brain regions Entorhinal cortex, hippocampus, superior frontal gyrus, and post-central gyrus. Analysis of the differentially expressed significant genes using Enrichr binned them into pathways. The top significant pathways involved riboflavin metabolism, neurodegenerative diseases such as Huntington’s disease, Amyotrophic lateral sclerosis, Parkinson’s disease, pathways of neurodegeneration, prion disease, and proteasome, endocytosis, and pathways involved in infection (Fig. 2a and Supplementary 1). Pathway analysis using upregulated genes showed allograft rejection, signalling, inflammatory, protozoan and cancer related pathways. Pathways obtained from downregulated genes include neurodegenerative diseases like HD, PD, AD, spinocerebellar ataxia, ALS and pathways associated with neurodegeneration. (Supplementary 2) The transcription factors involved include RUNX1, SMAD4, EGR1, GATAs, etc. (Supplementary 1 and 5). Further analysis of kinases involved CREB1, USF1, MYC, ATF2, YY1, etc. (Supplementary 4 and 5). The cell types involve glutamatergic and GABAergic neurons, CD4 + T-cells, microglia, and dendritic cell (Fig. 2b and Supplementary 6).

### GSE140829

Dataset was obtained from peripheral blood. Analysis of the differentially expressed significant genes using Enrichr binned them into pathways. The top significant pathways involved infection-based pathways like salmonella infection, Yersinia infection, Shigellosis, pathogenic Escherichia coli infection, signaling pathways such as NOD-like receptor signaling pathway, carbohydrate metabolism pathways like pentose phosphate pathway, and osteoclast differentiation (Fig. 2c and Supplementary 1). Pathway analysis using upregulated genes showed signalling, infection and osteoclast differentiation pathways. Pathways obtained from downregulated genes include ribosome, spliceosome, ubiquitin mediated proteolysis, circardian rhythm and metabolic pathways. (Supplementary 2) The transcription factors involved E2F1, USF2, RCOR1, SPI1, etc. (Supplementary 3 and 5). Kinase analysis showed the involvement of CDKs, MAPKs, CSNK2A1, and CK2ALPHA (Supplementary 4 and 5). The cell types affected include CD4 + T-cells, microglia (perivascular macrophage), CD4 + cytotoxic T-cell, and natural killer cells (Fig. 2d and Supplementary 6).

### Overlap of pathways, kinases, transcription factors, and cell types shows deregulated pathways and dysfunction of cell type with implications for disease

Further, we studied if the pathways obtained from different data sets across populations, samples, and study settings exhibit a common trend. The overlapping pathways include retrograde endocannabinoid signaling and salmonella infection are found in 4 datasets and Alzheimer’s disease, TCA cycle, endocytosis, pathways of neurodegeneration and proteasome occurred in 3 datasets while dopaminergic synapse and axon guidance are found in two of the datasets (Fig. 2e).

For understanding common transcription factors, we looked at the overlap of transcription factors from 5 datasets. Our study showed that 6 transcription factors are common among the different transcriptomic datasets. These transcription factors include CREB1, MYC, RCOR1, TCF3, UBTF, and ZNF384. Functional annotation of these TFs shows that they belong to signaling pathways, pathways related to infection, and protein synthesis pathways (Fig. 2f).

Further, we studied the kinases that are common among the different transcriptomic data sets. Analysis using the top 15 kinases from the 5 transcriptomic data sets conjured 10 kinases to be common among them. These kinases include AKT1, CDC2, CDK1, CDK2, CDK4, CK2Alpha, CSNK2A1, GSK3B, MAPK1, and MAPK14. Functional annotation of these kinases shows that they largely fall into those regulating cell proliferation, inflammation, etc. However, commonality analysis of kinases among the 5 kinases data sets using all the significant kinases conjured 69 kinases (Fig. 2g).

To corroborate the gene expression profile to different cell types that are associated with the disease we carried out a cell type analysis. We further looked at the overlap of the cell types obtained from the 5 transcriptomic data sets showed GABAergic neurons and Glutamatergic neurons to be common to 4 data sets, microglia to 3 of the data sets while T cells and Monocytes to two of the data sets (Fig. 2h). Corroborating the pathways, TFs, Kinases, and Cell type from commonality studies might shed light on the mechanisms associated with disease and factors that might modulate disease progression.

### Analysis of proteomic data sets from the literature shows pathways with potential implications for inflammation and disease progression

**Figure 3:**
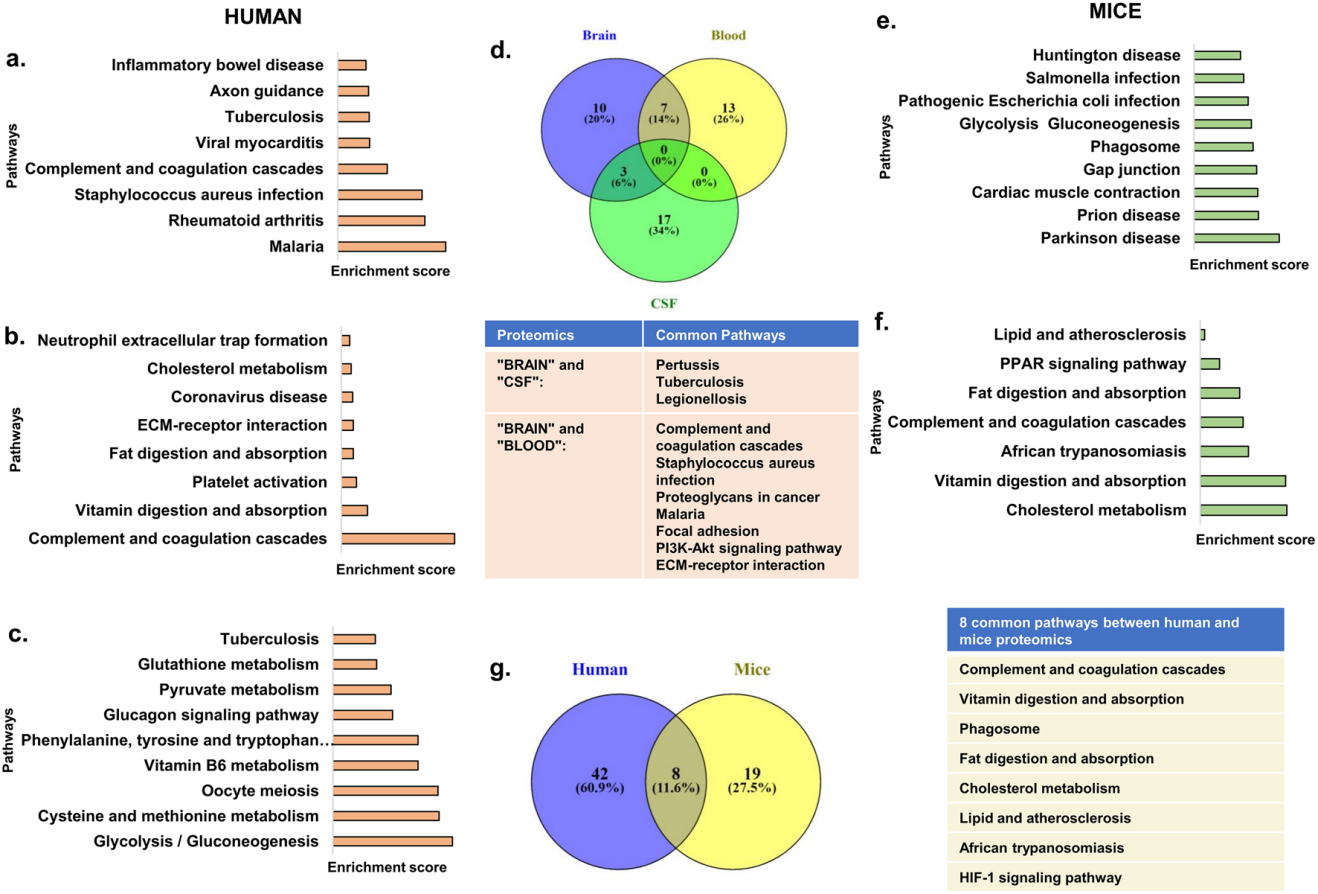
Pathway analysis of differentially expressed proteins (DEPs) between healthy controls and Alzheimer’s patients using Enrichr tool. Top significant pathways from datasets of (**a**). Brain (**b**). Blood serum (**c)**. Cerebrospinal fluid (CSF) (**d)**. Common proteomics pathways in humans. Proteomics analysis of mice datasets (**e).** Brain (**f)**. Blood (**g).** Common proteomics pathways between humans and mice. Figure generated using Venny (bioinfogp.cnb.csic.es/tools/venny/), Microsoft power point 2019 MSO (Version 2212 Build 16.0.15928.20196) 64-bit.

Proteomic analysis of brain^60^ data set from literature was performed using Enrichr using significantly differential proteins. Our analysis shows Fluid sheer stress and atherosclerosis, complement and coagulation pathway, various inflammatory diseases like systemic lupus erythematosus, inflammatory bowel disease, Rheumatoid arthritis, and infectious diseases like viral myocarditis, malaria, and tuberculosis (Fig. 3a and Supplementary 7). Proteomic analysis of the blood^61^ data set shows focal adhesion, ECM Receptor interaction, complement and coagulation pathway, infection disease pathways like staphylococcus aureus infection and coronavirus disease. Metabolic pathways like fat and vitamin digestion and absorption, Cholesterol metabolism, inflammatory process like neutrophil extracellular trap formation, and platelet activation were enriched (Fig. 3b and Supplementary 7). Proteomic analysis of the CSF^62^ data set shows enrichment of proteins belonging to Long Term Potentiation and infectious disease like glutathione metabolism. In particular, enrichment of metabolic pathways was observed in CSF. These major pathways include Glutathione and pyruvate metabolism, glycolysis/gluconeogenesis, Metabolism of amino acids (Methionine and cysteine, phenylalanine tyrosine, and tryptophan metabolism), and Vitamin B_6_ metabolism (Fig. 3c and Supplementary 7).

The commonality of the pathways among the different proteomic data sets shows complement and coagulation pathways, pathways enriched for various infectious diseases, phagosomes as well as signalling pathways. These pathways might have potential implications for inflammation and disease (Fig. 3d).

Further, we analysed the proteomic data sets from the brain and blood of mice models of Alzheimer’s disease^63,64^. Proteomic analysis of the brain^63^ data set showed pathways like Prion, Huntington’s and Parkinson’s disease, salmonella, pathogenic *E. coli* infection, phagosome and glycolysis, and gluconeogenesis (Fig. 3e and Supplementary 8). Proteomic analysis of the blood^64^ data set showed pathways like lipid and atherosclerosis, fat and vitamin digestion and absorption, Complement and coagulation pathway, and PPAR signalling (Fig. 3f and Supplementary 8).

We further looked for the pathways that are common to Alzheimer’s disease patients and mice models of disease. For the commonality studies, we pooled the respective patient and mice model pathway data sets and looked for overlapping pathways. Our analysis showed that a total of 8 pathways are common between the mice and human pathway data sets. The common pathways include complement and coagulation cascade, lipid and atherosclerosis, phagosome and HIF1 signalling, African trypanosomiasis, cholesterol metabolism and vitamin digestion and absorption are also found. The common pathways might be more important and representative of the disease as they are found to be conserved across taxa, populations, and study settings (Fig. 3g).

### Analysis of metabolomic data sets from the literature shows enrichment of pathways for immune-metabolism and disease progression

**Figure 4:**
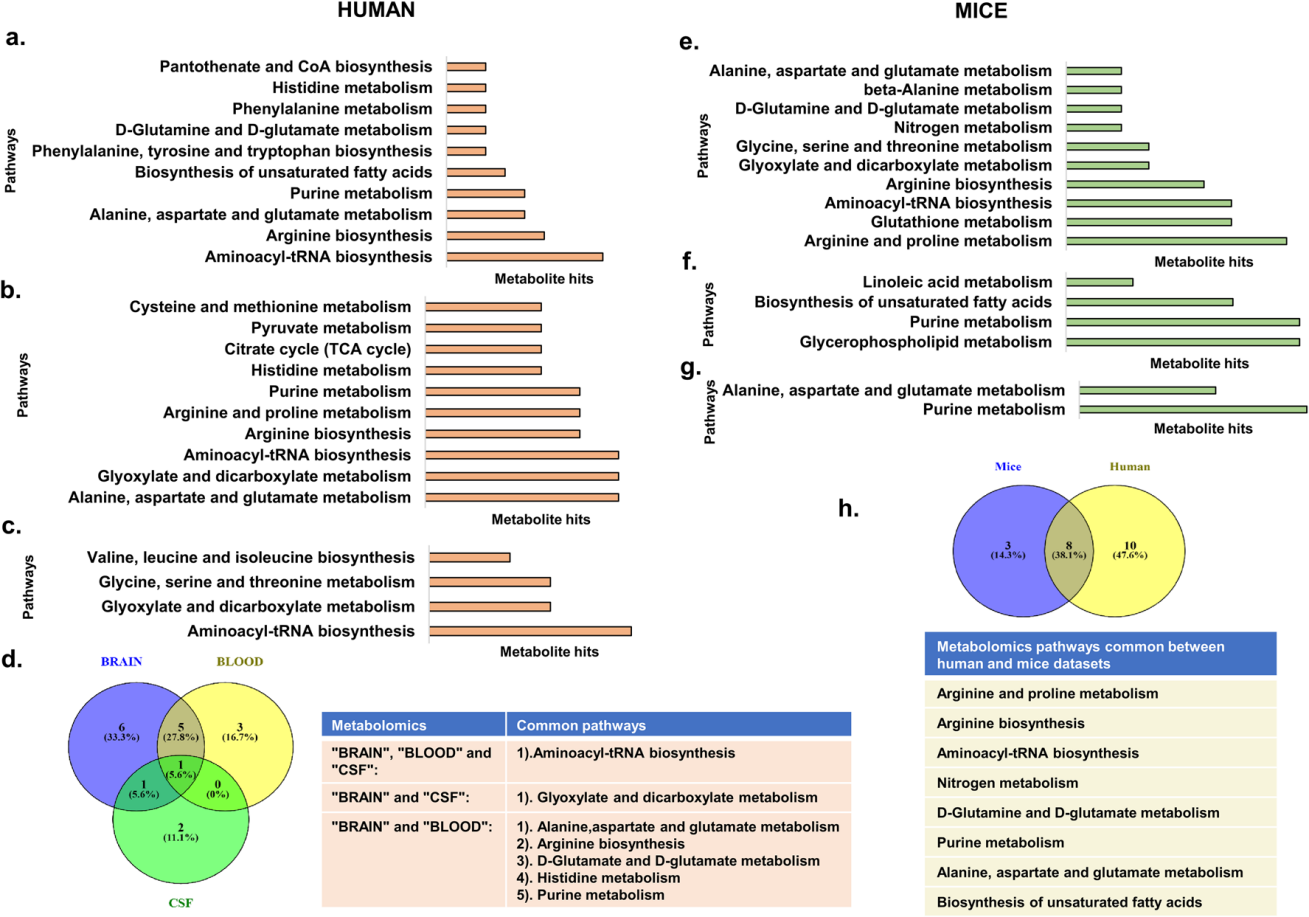
Pathway analysis of differential metabolites between healthy controls and Alzheimer’s patients using MetaboAnalyst tool (**a**). Brain (**b**). Blood serum (**c**). Cerebrospinal fluid (CSF) (**d**). Common metabolomic pathways in humans. Metabolomics analysis of mice datasets. (**e**). Brain (**f**). Blood (**g**). Cerebrospinal fluid (**h**). Common metabolomic pathways between humans and mice. Figure generated using Venny (bioinfogp.cnb.csic.es/tools/venny/), Microsoft powerpoint **2**019 MSO(Version 2212 Build 16.0.15928.20196) 64-bit.

Analysis of metabolomic data sets from the brain, blood, and cerebrospinal fluid was essentially performed using Metaboanalyst^65^ with significant differential metabolites obtained from the literature. Metabolomic analysis of brain^66^ data sets from the literature shows that Pantothenate and CoA biosynthesis, pyruvate metabolism, TCA Cycle, Glyoxylate and dicarboxylate metabolism, Purine metabolism, aminoacyl tRNA synthase, and metabolism of various amino acids (cysteine and methionine, histidine, arginine and proline, alanine aspartate and glutamate), as well as unsaturated fatty acids and arginine biosynthesis, are deregulated (Fig. 4a and Supplementary 9). Many of these metabolic pathways represent immune metabolism which is consistent with inflammation in Alzheimer’s disease. Analysis of blood^67^ metabolomic data sets showed deregulated pathways like pyruvate metabolism, TCA Cycle, Glyoxylate and dicarboxylate metabolism, Purine metabolism, aminoacyl tRNA synthase, and metabolism of various amino acids (cysteine and methionine, histidine, arginine and proline, alanine aspartate and glutamate) as well as arginine metabolism (Fig. 4b and Supplementary 9). Further, analysis of CSF^68^ metabolomic data sets showed pathways like aminoacyl tRNA biosynthesis, Valine, leucine and isoleucine biosynthesis, glycine, serine, and threonine metabolism as well as glyoxylate and dicarboxylate metabolism (Fig. 4c and Supplementary 9).

Commonality analysis of pathways obtained for the different human metabolomic data sets showed aminoacyl-tRNA biosynthesis to be common among them. The brain and CSF showed glyoxylate and dicarboxylate pathway to be common between them. The brain and blood data sets showed 5 common pathways which include alanine, aspartate and glutamate metabolism, glutamine and glutamate metabolism, histidine, and purine metabolism as well as arginine biosynthesis (Fig. 4d).

Similarly, we carried out the pathway analysis of metabolic data sets from the brain, blood, and cerebrospinal fluid from mice using metaboanalyst. The analysis for brain^69^ sample data sets showed deregulation of pathways like aminoacyl-tRNA and arginine biosynthesis, metabolism of alanine, aspartate and glutamate, Beta-alanine, glutamine and glutamate, nitrogen, glycine, serine and threonine, glyoxylate and dicarboxylate, glutathione as well as arginine and proline (Fig. 4e and Supplementary 10). Analysis of metabolic data sets from blood^70^ shows deregulation of linolenic acid, purine, and glycerophospholipid metabolism as well as biosynthesis of unsaturated fatty acid (Fig. 4f and Supplementary 9). Analysis of metabolic data sets from CSF^71^ also showed deregulation of Alanine, aspartate, glutamate, and purine metabolism (Fig. 4g and Supplementary 9).

Commonality studies was performed on the pooled pathways obtained for the human and mice metabolomic data sets. Our analysis showed that 8 pathways were common to the two data sets (Fig. 4h). The commonly deregulated pathways include Biosynthesis of aminoacyl-tRNA, arginine, unsaturated fatty acids and metabolism of nitrogen, Arginine and proline, glutamine and glutamate, purine as well as alanine, aspartate, and glutamate. The results suggest considerable concordance in metabolism between Alzheimer’s patients and mice models of disease despite differences in population, taxa, samples, and study settings.

**Figure 5:**
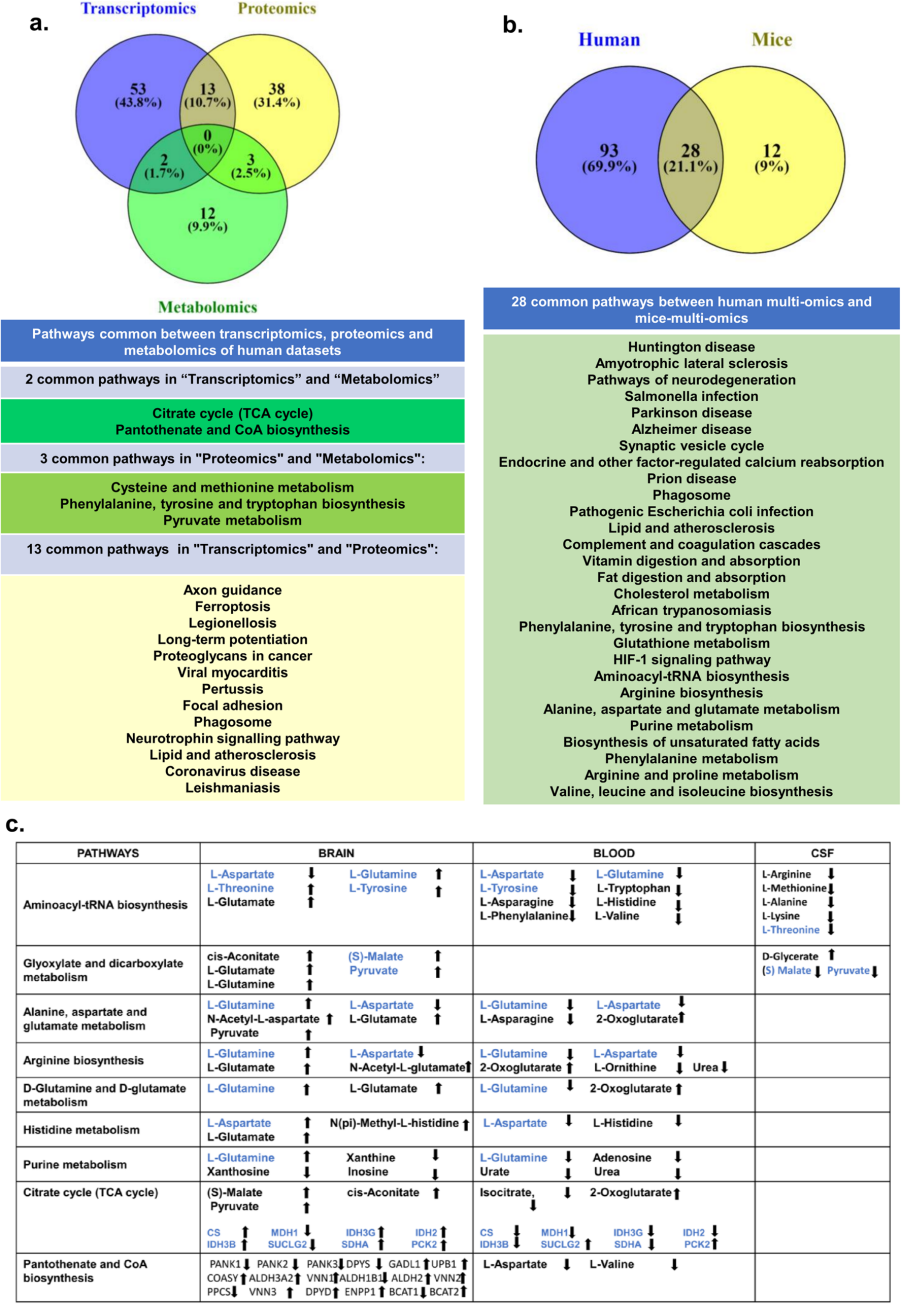
(**a**) Common pathways between transcriptomics proteomics and metabolomics from human datasets. (**b**) Common pathways between mice multi-omics data and human multi-omics data. (**c**) Metabolite expression levels of common metabolic pathways in humans. Metabolite and gene expression levels pathways common between transcriptomics and metabolomics in humans. Figure generated using Venny (bioinfogp.cnb.csic.es/tools/venny/), Microsoft powerpoint 2019 MSO (Version 2212 Build 16.0.15928.20196) 64-bit.

Further, we looked at the levels of metabolites in the pathways that are common to brain, blood and CSF. Previous studies have shown that the levels of different metabolites in brain bears an inverse correlation with those in the blood^72^. Consistent with this our analysis recapitulates these findings despite these are different data sets (Fig. 5c). In addition, from pathway obtained from transcriptomic data sets, we have also provided the expression profiles of these genes along with the metabolites in blood and brain (Fig. 5c).

### Commonality studies of multi-omics data sets shows the considerable overlap of pathways among the different data sets across taxa, populations, study settings, and platforms

The pooled transcriptomic human data sets show 100 pathways while the pooled proteomic data sets show 60 pathways and the pooled metabolomic data sets had 28 pathways. A total of 12 pathways were common to transcriptomics and proteomics (Fig. 5a) while 4 pathways were common between proteomics and metabolomics (Fig. 5a). The transcriptomic data set and metabolomic data set had two common pathways (Fig. 5a). Overall, the data sets show deregulation of inflammation and cytokine signalling pathways, metabolic pathways, neuronal signalling, etc. The cell type analysis also corroborates with the deregulated pathways observed in our multi-omic analysis. The transcription factor and kinase analysis shows critical regulators of these deregulated pathways.

The commonality studies of the pooled human and mice proteomic data sets showed 8 pathways were common to them (Fig. 3g). The commonality studies of the pooled human and mice metabolomic data sets showed 8 pathways between the data sets (Fig. 4h). The commonality studies of pooled human and mice pathways together showed 28 pathways are common (Fig. 5b). Taken together, our analysis captures the common pathways which are deregulated across taxa, populations, study settings, and different platforms used. These conserved pathways might have major ramifications for the observed symptoms, disease progression, and prognosis.

### Vitamin B_2_, B_5_, and B_6_ cofactor-protein interaction network pathway analysis showed considerable overlap with deregulated metabolic pathways in Alzheimer’s disease patients and mice models

**Figure 6:**
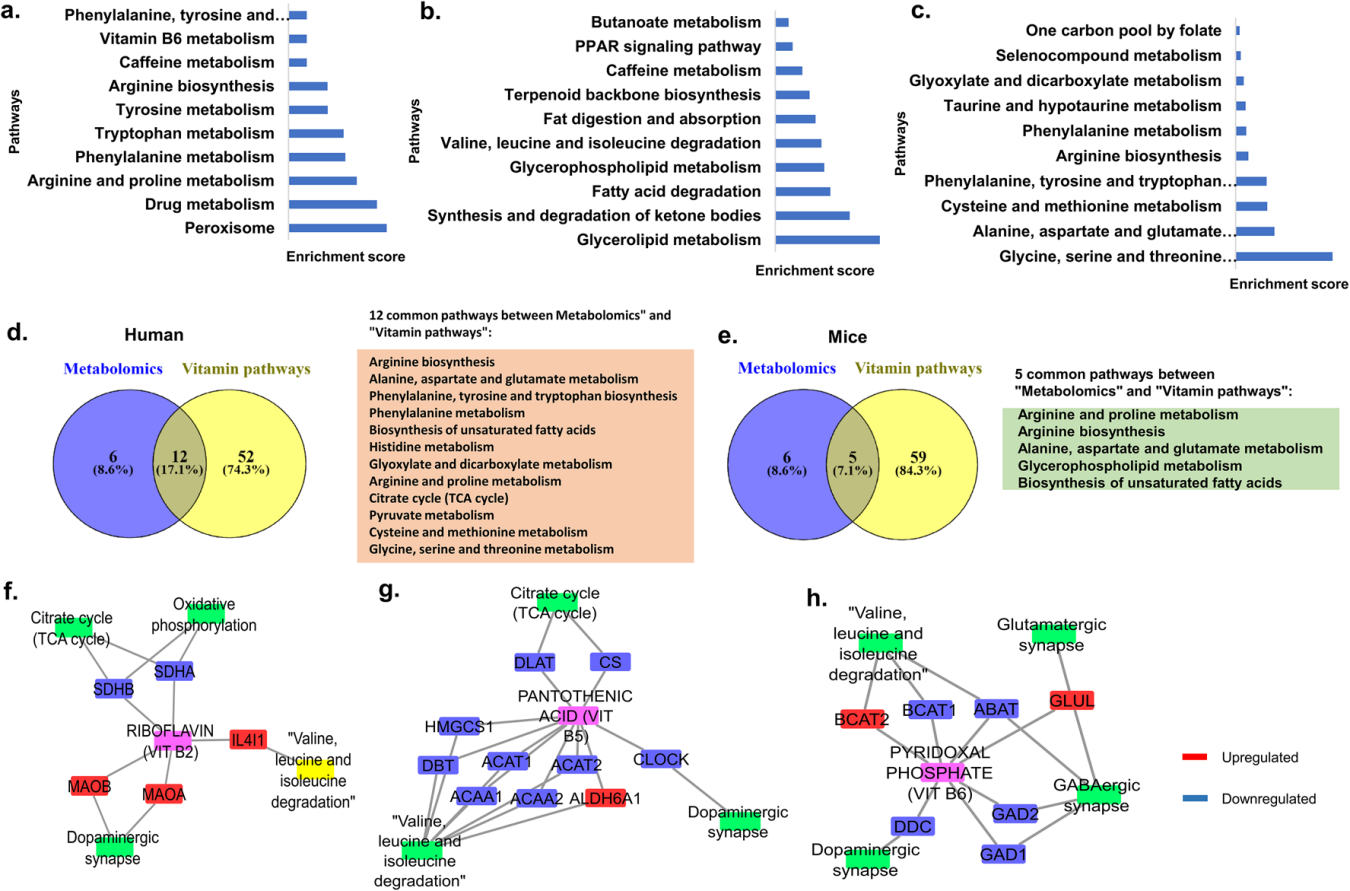
Pathway analysis of vitamin co-factor network. Top significant pathways from (**a**)**.** Vitamin-B_2_ (**b)**. Vitamin-B_5_ (**c**). Vitamin-B_6_. Commonality analysis (**d**). Pathways common between human metabolomics and pooled vitamin-cofactor analysis (**e**). Pathways are common between mice metabolomics and pooled vitamin co-factor analysis. Network representing cofactor network of (**f**). B_2_ with metabolomic pathways. (**g**). B_5_ with metabolic pathways (**h**). B_6_ with metabolic pathways. Figure generated using Microsoft powerpoint 2019 MSO(Version 2212 Build 16.0.15928.20196) 64-bit, cytoscape (cytoscape 3.8.2) cytoscape/cytoscape/3.8.2/. Venny (bioinfogp.cnb.csic.es/tools/venny/).

Integrated multi-omics analysis showed the deregulation of Vitamin B_2_, B_5_, and B_6_ pathways. Hence, we constructed the Vitamin B_2_, B_5_, and B_6_ protein interactions network and binned the genes into pathways (Fig. 6a–c and Supplementary 11). Commonality studies of human (Fig. 6d) and mice metabolic pathways (Fig. 6e) with significant pathways from vitamin cofactor analysis showed considerable overlap. The transcriptomic data was further imported into the network. Vitamin B_2_ is a cofactor for succinate dehydrogenase complex flavoprotein subunit A (SDHA), and succinate dehydrogenase complex iron sulphur subunit B (SDHB) that are involved in the citric acid cycle and oxidative phosphorylation (Fig. 6f). Pantothenate is a precursor for Coenzyme A. The Valine, leucine, and isoleucine degradation pathway is deregulated in Alzheimer’s disease. Various genes associated with this pathway such as Acetyl-Coenzyme A Acetyltransferase 2 (ACAT2), acetyl-CoA acetyltransferase 1 (ACAT1), 3-Hydroxy-3-Methylglutaryl-CoA Synthase 1 (HMGCS1), acetyl-Coenzyme A acyltransferase 1 (ACAA1), dihydrolipoamide branched chain transacylase E2 (DBT), Acetyl-CoA Acyltransferase 2 (ACAA2) are down-regulated while Aldehyde Dehydrogenase 6 Family Member A1 (ALDH6A1) is upregulated. The enzyme which utilizes vitamin B_2_ as a cofactor, Interleukin-4-Induced Protein 1 (IL4I1) an L-amino acid oxidase, and monoamine oxidases (MAOA and MAOB) are upregulated while SDHA and SDHB are downregulated (Fig. 6g).

B6-dependent enzyme Dopa decarboxylase (DDC) in the dopaminergic synapse, Glutamate decarboxylases (GAD1, GAD2) in GABAergic synapse, ABAT and BCAT1 in valine, leucine, and isoleucine degradation pathway are down-regulated. The enzymes Glutamine synthetase (GLUL) in glutamatergic synapse and GABAergic synapse is upregulated while the BCAT2 is upregulated in the valine, leucine, and isoleucine degradation pathway (Fig. 6h).

**Figure 7:**
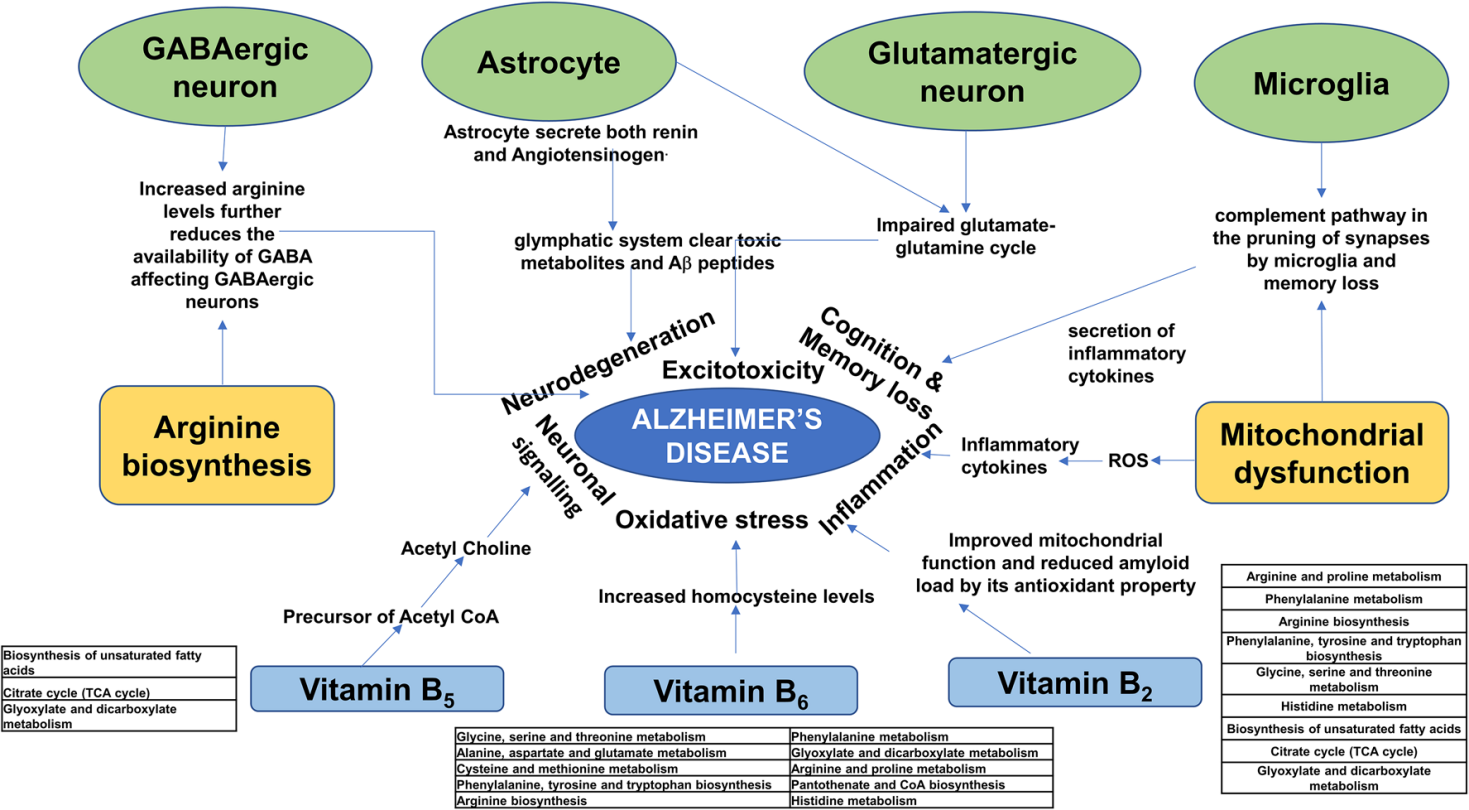
Summary of overall findings from multi-omics studies. Role of vitamin B_2_, B_5_ and B_6_ modulated pathways with overlap with deregulated metabolic pathways in AD and its implications for observed symptoms. Cell types affected and their role in symptoms associated with AD. Deregulated energy metabolism and their effect on AD symptoms. Figure generated using Microsoft power point 2019 MSO (Version 2212 Build 16.0.15928.20196) 64-bit.

Overall, our multi-omics study results are well correlated with the signs and symptoms of AD. AD is associated with memory loss, cognition, inflammation, oxidative stress, synaptic dysfunction and neuronal dysfunction. Figure 7 and Supplementary file 14 depicts the involvement of pathways, transcription factors, kinases and cell types and pathways associated with each cell type in AD from our multi-omics analysis.

## Discussion

### Implications of deregulated pathways from transcriptomic datasets to AD

Our integrated multi-omics analysis showed pathways related to neuronal signalling. The deregulated pathways include dopaminergic, cholinergic synapses, nicotine, and morphine addiction, and choline metabolism in cancer which are all downregulated. We also find that the B_6_ pathway is deregulated in the proteomic data sets. Previous studies have shown low levels of B_6_, B_12_, or folate lead to elevated levels of Aβ aggregates in neurons of transgenic mice models of Alzheimer’s disease^30,31,73^. B_6_ is a cofactor that is involved in dopaminergic, and GABAergic synapse function^74^. The cholinergic synapses are also deregulated in Alzheimer’s disease^75^. The choline pathway is dependent on the biosynthesis of choline which in turn is dependent on the methionine pathway^31^. In addition, acetylcholine biosynthesis is also dependent on the levels of acetyl CoA. However, our transcriptomic and metabolomic analysis also shows that pantothenate pathway is deregulated. Pantothenate (B_5_) is an important substrate in CoA biosynthesis. The levels of CoA decide the availability of acetyl CoA for the biosynthesis of acetylcholine^76^. Our analysis clearly captures the role of B_6_ and pantothenate in the deregulation of neuronal signalling and function. In addition, proteomic and metabolomic analysis shows deregulation of cysteine and methionine metabolism. Methionine is an important component involved in the biosynthesis of choline^32^. In the methionine cycle the by-product homocysteine is either converted into methionine in a pathway involving B_12_ or folate or into cysteine through the trans-sulphuration pathway involving B_6_^30^. Cysteine is an important substrate used in the biosynthesis of cellular antioxidants like glutathione, taurine, coenzyme A and lanthionine^77^. Reduced B_6_ will lead to reduced levels of cysteine. Consistent with these observations, glutathione pathway was found to be deregulated in proteomic analysis. Elevated oxidative stress is associated with AD, which contributes to disease progression^78–80^.

### B_2_, B_5_ and B_6_ modulate deregulated metabolic pathways in AD

Our analysis also showed impaired riboflavin (B_2_) metabolism. Previous studies have shown that B_2_ metabolism is affected in AD^37^. Using a yeast model it was demonstrated that riboflavin improved mitochondrial function and reduced amyloid load in AD^81^. To further delineate the role of vitamins/cofactors like riboflavin, B_6_, and pantothenate in metabolic deregulation and AD we generated a vitamin/cofactor-protein interaction network. The interacting proteins were further binned into pathways. The pathways so obtained were then analysed for overlap with deregulated pathways obtained from the multi-omics analysis of AD data sets. Considerable concordance in pathways was observed between the two data sets. Taken together, our results show good concordance between neuronal dysfunction with metabolic pathways. In particular, our results show a potential role for riboflavin (B_2_), B_6_, and pantothenate in the disease process.

Water-soluble vitamins are coenzymes for enzymes involved in metabolic pathways involved in cellular functions. Poor vitamin B_6_ status is commonly observed among older people and higher amounts of homocysteine levels have been recommended as the cause of this deficiency^82^. Supplementation with B vitamins including B_6_ has been shown to decrease blood homocysteine levels and slow down the atrophy of specific brain regions^38^. Severe cerebral deficiency of vitamin B_5_ is observed in the AD brain. Vitamin B_5_ is the obligate precursor of acetyl-coenzyme-A which has a wide range of functions, it is the precursor of the neurotransmitter acetylcholine^76^. CoA is also essential for many metabolic process like TCA cycle^83^. Mice studies indicate vitamin B_2_ protects AD-affected brain from ROS-induced damage, probably due to its anti-oxidant property^37^. So as a new disease-modifying strategy vitamin supplementation is widely being explored^70^.

### Deregulated arginine pathway and its implications for AD

Previous studies have shown that AD is associated with metabolic remodelling. Analysis of metabolomic data sets of AD brain and blood from literature^84^ shows deregulation of arginine biosynthesis. Arginine is an important metabolite for the biosynthesis of Nitric oxide by NOS^85^. Further, increased arginine is also indicative of arginosuccinate a precursor in arginine biosynthesis which is associated with Guinidilation of GABA to give guanidinobutanoic acid^86^. The production of guanidinobutanoic acid further reduces the availability of GABA, which is already low due to low B_6_ levels leading to GABAergic neuron dysfunction^31^. Indeed, cell type analysis from transcriptomic data sets showed the involvement of GABAergic neurons.

### Mitochondrial dysfunction, metabolic remodelling and ROS: implications for AD

Our multi-omic analysis revealed AD is associated with mitochondrial dysfunction as well as deregulation of pathways like pyruvate metabolism, TCA cycle and nucleotide metabolism, and glutamine and glutamate metabolism. Previous studies in yeast model of ALS has shown that either inhibition or knock out (KO) of genes in complex III and IV lead to clearance of protein aggregates^87^. mice model of AD has shown that KO of complex III gene led to reduce a-beta aggregation^88^. Similarly, KO of genes involved in mitochondrial fission helped to clear protein aggregates in yeast model of HD^89^. Activation of immune cells leads to metabolic remodelling^90^. The mitochondrial function is impaired and the cells rely on activation of glycolysis, TCA cycle, glutaminolysis, and nucleotide metabolism^91^. Studies have shown that the increased intermediates in glycolytic and glutaminolysis pathways are pooled for the growth and proliferation of immune cells^92^. In addition, changes in phenylalanine which is associated with inflammation are also associate with AD^47^.

Mitochondrial dysfunction leads to elevated levels of ROS and oxidative stress^93^. In addition, the inflammatory response also leads to elevated levels of ROS. ROS is shown to mediate signalling processes leading to the expression of inflammatory cytokines^94,95^. Scavenging of ROS is shown to attenuate inflammation. Consistent with inflammation analysis of transcriptomic data sets from the GEO database shows enrichment of TNF signalling pathway in AD. The results of this analysis suggest elevated levels of cytokines, ROS, oxidative stress, immune metabolism and involvement of immune cells in AD.

### The role of cell types in AD progression

Our cell type analysis also shows changes in immune cell types like T cells, microglia, astroglia, etc. Renin-angiotensin system regulates the blood pressure and fluid balance and in brain it is involved in memory acquisition and consolidation^96^. In CNS astrocytes synthesis and secrete both renin and Angiotensinogen^97^. The renin-angiotensin system is involved in the functioning of glymphatic system and helps to clear toxic metabolites and Aβ peptides^98^. Hypertension decreases CSF flow in the glymphatic system thus adversely affecting clearance of toxic metabolites or Aβ peptides resulting in it aggregation, contributing to neurodegeneration^99^. Further, studies have shown that inhibition of immune metabolism lead, to impaired synthesis and secretion of inflammatory cytokines by microglia^100^. Taken together our analysis shows activation of immuno-metabolism in AD. The glutamatergic dysfunction is associated with AD^101^. In brain, the glutamate from the synapse is taken through the excitatory amino acid transporters (EAAT) by astrocytes. Glutamate is further converted to glutamine and it is released by astrocytes which is taken up by the neurons^102^. AD is shown to have impaired glutamate-glutamine cycle resulting in excitotoxicity and death of neurons^103^. The GABAergic neurons are considerably reduced in AD compared to controls^104^. Excess amounts of Aβ downregulate GABA inhibitory interneuron activity that cause loss of inhibitory interneurons^104^. Further, our analysis shows the deregulation of complementation and coagulation pathway. Interestingly studies have shown the role for complement pathway in the pruning of synapses by microglia and memory loss^105^. The complement protein was shown to label the synapse to be pruned which is subsequently acted upon by the microglial cells^106^. The upregulation of complement pathway concomitant with microglial activation due to the inflammatory milieu in AD might prove to be detrimental to the disease process^107^. The impinging evidence thus accounts for the observed memory loss during disease progression in AD.

### Implications of the findings for AD pathology and management

The study shows an important role for metabolic deregulation of pathways like TCA, Arginine, glutathione and energy metabolism, cell types like microglia, astroglia and neuronal cell types, Vitamin B_2_, B_5_ and B_6_ ROS and oxidative stress in AD. Previous studies using MRI have shown that atrophy of affected regions in the brain of genetically susceptible individuals precedes the onset of symptoms in AD^17^. Neuronal protection early during the pre-symptomatic stage in genetically susceptible individuals could help to prolong the onset of symptoms in AD. Further, incorporation of N-acetyl cysteine or other anti-oxidants in the diet might also help to mitigate oxidative stress and neuronal cell death in AD^108^. Dietary intervention with B_2_, B_5_ and B_6_ will help to achieve favourable prognosis in AD. Overall, our results show that a combination of antioxidants, B_2_, B_6_, and pantothenate (B_5_) during the pre-symptomatic stage in genetically susceptible individuals might help to postpone the onset of symptoms and potentially help in better management of AD. Above all this study will help to appreciate the role of deregulated pathways in the biology of AD and as potential therapeutic targets to manage the disease. Summary of the findings from this study are shown in Fig. 7 and detailed information is provided in Supplementary 14.

## Methods

### Transcriptomics data analysis

Transcriptomics data from previously published literature curated in Gene Expression Omnibus (GEO) database was searched for Alzheimer’s disease and only those GEO datasets were selected where the differential gene expression analysis could be done by the GEO2R tool for gene expression data. We have analysed the gene expression datasets with GSE IDs—GSE5281^52^, GSE36980^53^, GSE44770^54^, GSE48350^55^, GSE140829^56^. Single cell transcriptomics examines the gene expression levels of individual cell types in a given population. The transcriptomic data sets used in this study are from those which represent only controls and AD patients. We have also ensured that the data sets have enough subjects for analysis and comparison. Detailed information of age, number, and gender of subjects is provided in Supplementary file 12.

### GEO2R analysis

The GEO2R tool was used to compare two or more sample groups in a GEO dataset to find differentially expressed genes (DEGs) in various experimental or clinical conditions. The samples to be evaluated are listed in the GEO2R^109^ analysis tool, and the samples are divided into two or more experimental or clinical groups. When comparing test samples to controls, the fold change between the groups follows the convention of positive for genes upregulated and negative for genes downregulated. Running the GEO2R analysis with default parameters after the samples have been assigned to the groups yields fold change in the top DEGs between the test and control groups based on their p-value. The DEGs were sorted based on the adjusted p-value for further analysis when the findings were downloaded. For subsequent analysis, differentially expressed genes having an adjusted *p*-value ≤ 0.05 were chosen. Upregulated and downregulated genes were segregated based on fold change value and pathway and transcription factor analysis was performed separately.

### Gene enrichment analysis by Enrichr

For the list of differentially expressed genes (with an adjusted *p*-value ≤ 0.05) that were found during GEO2R analysis, gene enrichment analysis was performed using Enrichr maayanlab.cloud/Enrichr/^57,58^. Enrichr examines these genes using databases such as the KEGG^110^, Wiki, and Reactome pathway databases, as well as the DisGeNet database and the Gene Ontology (GO) database for biological processes, to determine which enriched pathways, biological processes, and diseases do they belong. Enrichr uses the combined score to identify pathways and cell types. Multiplying the Z-score of the deviation from the expected rank by the log of p-value from Fisher's exact test, the combined score is calculated^58^. The results are presented as a bar graph containing pathways, biological processes, and diseases based on the combined score. Cell type analysis was performed for all datasets using the Azimuth database.

### iLINCS server (kinase and transcription factor analysis)

iLINCS^111^ is a web tool that allows studying gene expression data and signatures from GEO Datasets. From the input of gene expression data, this platform allows for the analysis of gene enrichment networks, associated transcription factors, and kinases. We used the iLINCS X2K maayanlab.cloud/X2K/ analysis tool to find the essential transcription factors and kinases engaged in or linked with differentially expressed genes in GEO datasets. Transcription factors and kinases with a hypergeometric *p*-value ≤ 0.05 are considered significant. Transcription factor analysis of upregulated and downregulated genes was performed separately and provided in Supplementary 2. The network analyst tool networkanalyst.ca/ was used to identify the potential pathways that these transcription factors and kinases are engaged in, as well as the target genes of these transcription factors and kinases.

### Proteomics data analysis

Proteomics analysis was performed for three tissues blood serum, brain cortex, and cerebrospinal fluid using Enrichr maayanlab.cloud/Enrichr/. The datasets were obtained from existing published literature. To understand the dynamics at the protein level in different tissues (1) Brain Cortex^60^: 58 differentially expressed significant proteins were obtained from a published dataset containing 100 Alzheimer’s samples to find the deregulated pathways. (2) Blood Serum^61^: 22 differentially expressed significant proteins with *p*-value < 0.05 and fold change(upregulated—1.5 and downregulated—0.67) were obtained from a published dataset with 20 Alzheimer’s patient samples and 20 control samples to find the deregulated pathways. (3). Cerebrospinal Fluid^62^: 65 differentially expressed significant proteins (63upregulated and 2 downregulated) with *p*-value < 0.0001 were obtained from a published dataset with 20 Alzheimer’s patient samples and 20 cognitively normal individuals samples to find the deregulated pathways. A detailed information of number of human subjects, gender and age is provided in Supplementary file 12.

The mice data selected had only proteomics and metabolomics. We also find that some of the data sets had very small number of metabolites which did not provide any significant pathways or overlap completely with pathways obtained from the present data set used in the study. The mice model is used to compare with human AD data sets to demonstrate that irrespective of different study settings, taxa and techniques used there is a considerable overlap of deregulated pathways among them. In addition, mice model is well appreciated and the overlapping pathways could be considered the core deregulated pathways that might be involved in disease progression. Mice proteomics analysis was performed for brain and blood tissues, datasets were obtained from the literature. The details of mice used for blood proteomics is triple transgenic mice 3xTg-AD mice expressing the human mutations APPSwe, PS1 M146V, and Tau P301L and strain B6129SF2/J. Details of mice used for brain proteomics is 3xTg AD mice and control strain used C57/BL6. Differentially expressed mice proteins are converted into human orthologs using DIOPT flyrnai.org/diopt and used for pathways analysis. Overall 148 significant differential proteins from the cortex and 93 hippocampal proteins are used for pathway analysis^63^. 17 significantly deregulated proteins from blood serum are used for pathway analysis^64^. Detailed information of number of mice, gender, age and strain of all mice datasets are provided in Supplementary file 12.

### Metabolomic data analysis

AD metabolomics studies give us insight into the pathophysiology of disease as the metabolites are collective termini of transcriptomics and proteomic profiles. We analysed three published datasets of AD patients obtained from the literature. Metaboanalyst 5.0 metaboanalyst.ca/ a web-based tool was used to analyse the pathways associated with the metabolites. Pathways with FDR (false discovery rate) ≤ 0.25 are considered significant and used for further analysis.

Metabolomic analysis was performed on three tissues. The datasets were obtained from published literature. (1). Brain Cortex^66^: significant metabolites with *p*-value ≤ 0.05 were obtained from a dataset containing 21 AD patients and 19 control samples (2). Blood Serum^67^: 16 decreased and 7 increased differential metabolite s with adj. *p*-value ≤ 0.05 were obtained from a published dataset with 23 AD and 21 control samples (3). Cerebrospinal fluid^68^: 54 significant differential metabolites were obtained from a published dataset with 10 control samples and 10 AD patient samples. A detailed information of number of human subjects, gender and age is provided in Supplementary file 12.

Mice metabolomics analysis was performed for the brain, blood and cerebrospinal fluid. The datasets were obtained from literature. Mice strain used for studies was C57BL/6J with APP/PS1 coexpressing the Swedish mutation(K595N/M596L) and the detaE9 with PS1 exon deletion in blood and brain and K670N, M671L mutations in CSF mice not expressing transgene was used as wildtype controls in blood, brain and CSF. A total of 73 significantly deregulated metabolites from brain are used for pathway analysis and^69^. 15 significantly deregulated metabolites from blood plasma of APP/PS1 are used for pathway analysis^112^. 20 significantly deregulated metabolites from cerebrospinal fluid are used for pathway analysis^71^. Detailed information of number of mice, gender, age and strain of all mice datasets are provided in Supplementary file 12.

### Commonality study

Common significant pathways, cell types, transcription factors and kinases from 5 datasets are identified using multiple list comparator tool molbiotools.com/listcompare.php. Pathways and cell types with *p*-value ≤ 0.05 are selected and further top 20 pathways and cell types with the highest combined score are selected for commonality study. Commonality study and venn diagram generation for proteomics and metabolomics datasets were carried out by venny online tool bioinfogp.cnb.csic.es/tools/venny/.

### Cofactor-protein interacting network and its overlapping genes with enriched pathways from transcriptomics datasets

A cofactor-protein interaction network for vitamin B_2_ (riboflavin), B_5_ (pantothenate), and B_6_ (pyridoxine) and its interacting proteins were generated using pre-published data in cytoscape 3.8.2^113,114^. A network was generated for each transcriptomic dataset. A comparative analysis was carried out for common genes between the cofactor-protein network and those involved in the enriched pathways from transcriptomic dataset analysis. Further, Cytoscape 3.8.2 was used to generate the integrated Transcriptomic-Cofactor-protein interaction network using the network merge tool for visualization. The generated network highlights key overlapping genes that are represented in both the cofactor-protein network and enriched pathway network from transcriptomics as shown.

## Supplementary Information

Supplementary Information 1.

Supplementary Information 2.

Supplementary Information 3.

Supplementary Information 4.

Supplementary Information 5.

Supplementary Information 6.

Supplementary Information 7.

Supplementary Information 8.

Supplementary Information 9.

Supplementary Information 10.

Supplementary Information 11.

Supplementary Information 12.

Supplementary Information 13.

Supplementary Information 14.

## Data Availability

All the transcriptomic datasets analysed in this study are taken from NCBI GEO (Gene Expression Omnibus) database which are already publicly available. Accession numbers for GEO datasets used in the study are GSE5281- (https://www.ncbi.nlm.nih.gov/geo/query/acc.cgi), GSE36980-(https://www.ncbi.nlm.nih.gov/geo/query/acc.cgi), GSE44770 (https://www.ncbi.nlm.nih.gov/geo/query/acc.cgi), GSE48350 (https://www.ncbi.nlm.nih.gov/geo/query/acc.cgi), GSE140829 (https://www.ncbi.nlm.nih.gov/geo/query/acc.cgi). All the proteomic and metabolomic datasets analysed during this study are taken from literature (published article and its supplementary files). The detailed information of all the datasets used in this study are given in Supplementary file 13 and appropriately referenced. No animals and humans are directly involved in the study.
