# Supplementary material for: Integrated multi-omics analysis of Alzheimer’s disease shows molecular signatures associated with disease progression and potential therapeutic targets
Source: Sci Rep. 2023 Mar 6;13:3695. doi: 10.1038/s41598-023-30892-6 (PMC9986671; doi:10.1038/s41598-023-30892-6)
Supplement: Supplementary file 2 — Supplementary Information 2. [file 41598_2023_30892_MOESM2_ESM.pdf]

Integrated multi-omics analysis of Alzheimer's disease shows molecular signature associated with disease progression and potential therapeutic targets

Pradeep Kodam[1], Sai Swaroop. R[2], Sai Sanwid Pradhan[2], Venketesh Sivaramakrishnan[2*], Ramakrishna Vadrevu[1*]

[1]Department of Biological Sciences, Birla Institute of Technology and Science Pilani, Hyderabad Campus, Jawahar Nagar, Hyderabad, 500078 Telangana, India

[2]Disease Biology Lab, Department of Biosciences, Sri Sathya Sai Institute of Higher Learning, Prasanthi Nilayam, Anantapur, 515134 Andhra Pradesh, India

[*]Corresponding author

* Venketesh Sivaramakrishnan: s.venketesh@gmail.com

* Ramakrishna Vadrevu: vrk@hyderabad.bits-pilani.ac.in

Keywords: Alzheimer's disease, Integrated multi-omics, Vitamin-cofactor analysis, mice model, neurodegenerative disease.

Supplementary-2 : Pathway and transcription factor analysis of upregulated and downregulated genes separately.

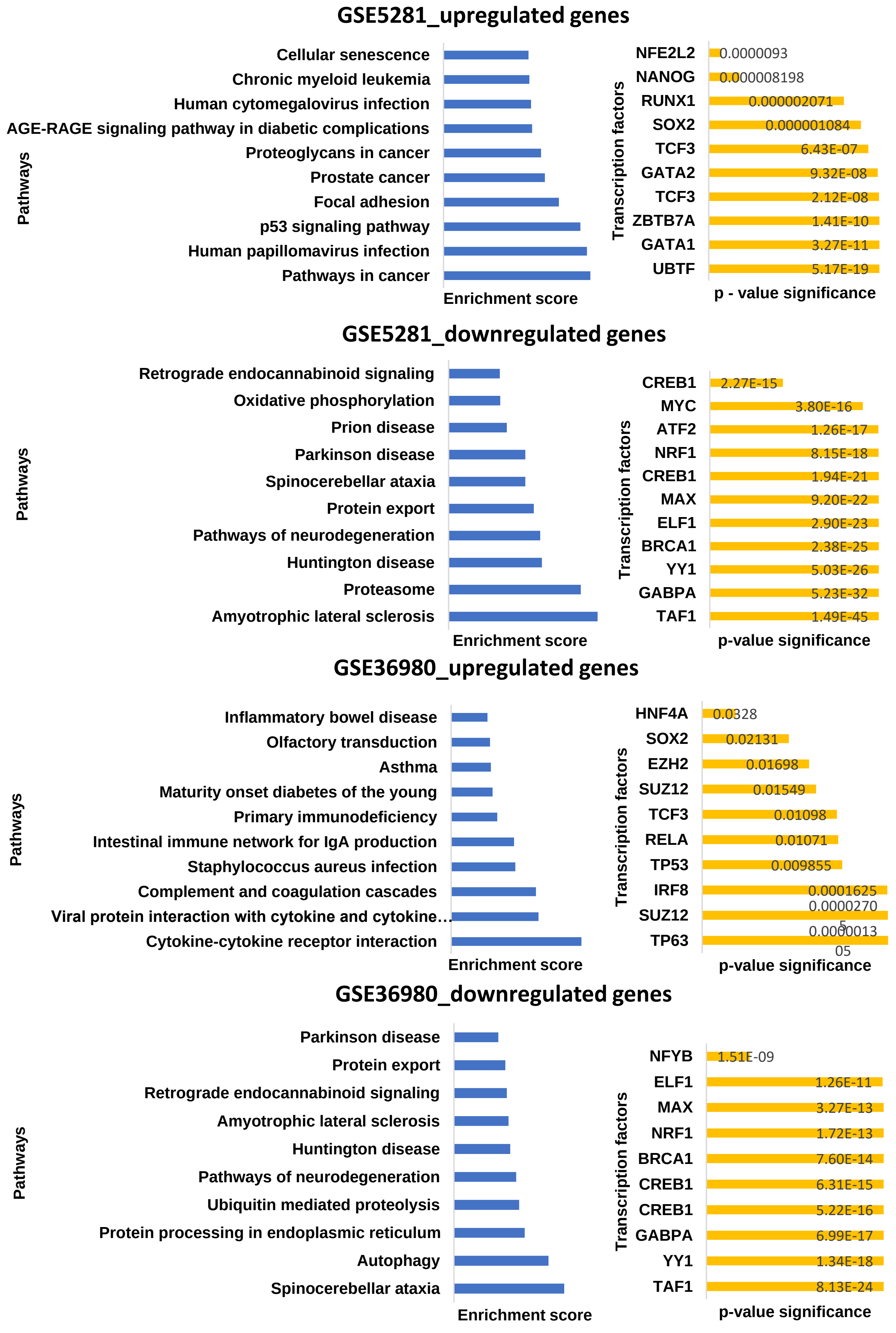
GSE5281_upregulated genes
Pathways
Cellular senescence
Chronic myeloid leukemia
Human cytomegalovirus infection
AGE-RAGE signaling pathway in diabetic complications
Proteoglycans in cancer
Prostate cancer
Focal adhesion
p53 signaling pathway
Human papillomavirus infection
Pathways in cancer
Enrichment score
Transcription factors
NFE2L2 0.0000093
NANOG 0.000008198
RUNX1 0.000002071
SOX2 0.000001084
TCF3 6.43E-07
GATA2 9.32E-08
TCF3 2.12E-08
ZBTB7A 1.41E-10
GATA1 3.27E-11
UBTF 5.17E-19
p - value significance
GSE5281_downregulated genes
Pathways
Retrograde endocannabinoid signaling
Oxidative phosphorylation
Prion disease
Parkinson disease
Spinocerebellar ataxia
Protein export
Pathways of neurodegeneration
Huntington disease
Proteasome
Amyotrophic lateral sclerosis
Enrichment score
Transcription factors
CREB1 2.27E-15
MYC 3.80E-16
ATF2 1.26E-17
NRF1 8.15E-18
CREB1 1.94E-21
MAX 9.20E-22
ELF1 2.90E-23
BRCA1 2.38E-25
YY1 5.03E-26
GABPA 5.23E-32
TAF1 1.49E-45
p-value significance
GSE36980_upregulated genes
Pathways
Inflammatory bowel disease
Olfactory transduction
Asthma
Maturity onset diabetes of the young
Primary immunodeficiency
Intestinal immune network for IgA production
Staphylococcus aureus infection
Complement and coagulation cascades
Viral protein interaction with cytokine and cytokine…
Cytokine-cytokine receptor interaction
Enrichment score
Transcription factors
HNF4A 0.0328
SOX2 0.02131
EZH2 0.01698
SUZ12 0.01549
TCF3 0.01098
RELA 0.01071
TP53 0.009855
IRF8 0.0001625
SUZ12 0.0000270
5
TP63 0.0000013
05
p-value significance
GSE36980_downregulated genes
Pathways
Parkinson disease
Protein export
Retrograde endocannabinoid signaling
Amyotrophic lateral sclerosis
Huntington disease
Pathways of neurodegeneration
Ubiquitin mediated proteolysis
Protein processing in endoplasmic reticulum
Autophagy
Spinocerebellar ataxia
Enrichment score
Transcription factors
NFYB 1.51E-09
ELF1 1.26E-11
MAX 3.27E-13
NRF1 1.72E-13
BRCA1 7.60E-14
CREB1 6.31E-15
CREB1 5.22E-16
GABPA 6.99E-17
YY1 1.34E-18
TAF1 8.13E-24
p-value significance

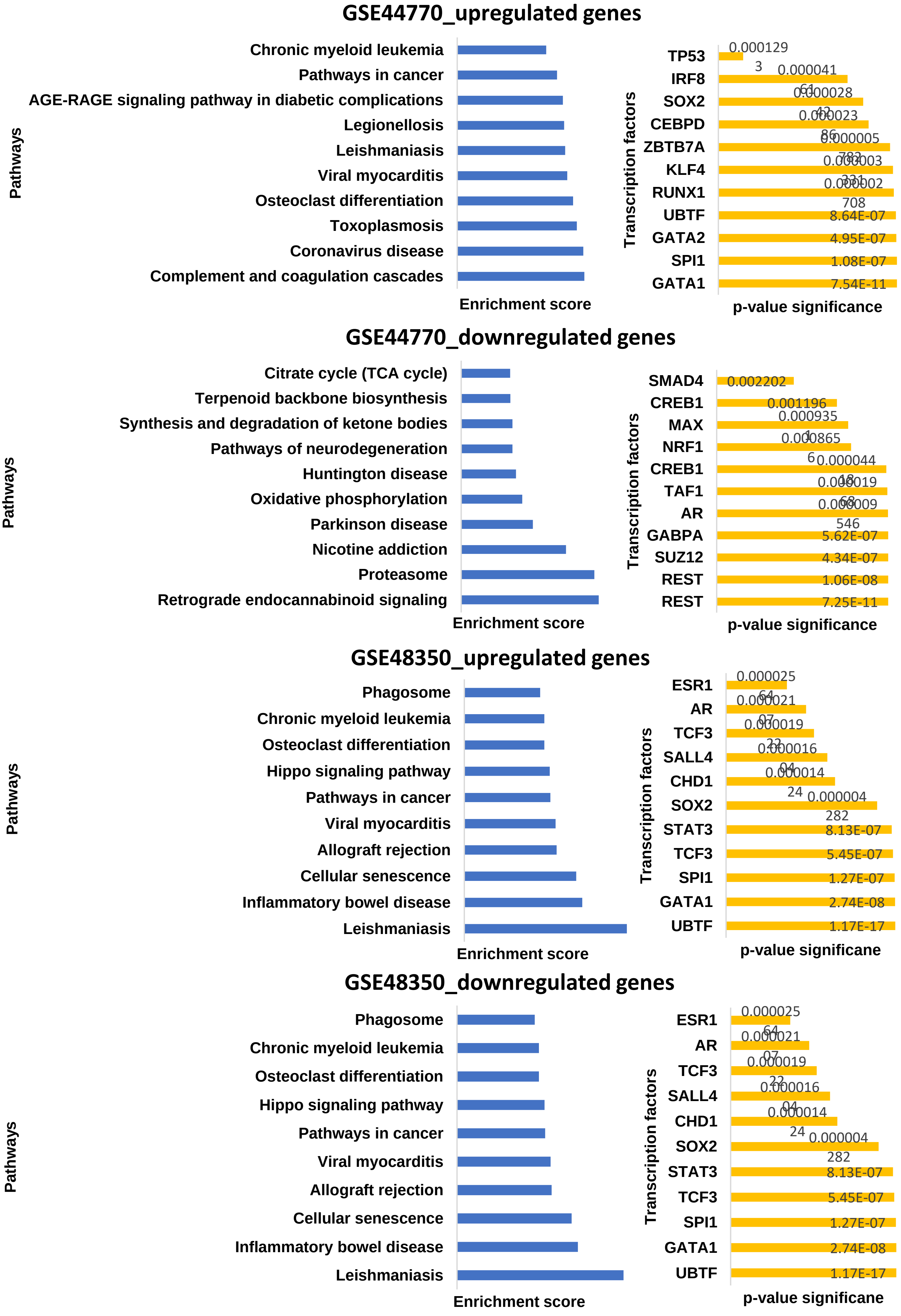
GSE44770_upregulated genes
Pathways
Chronic myeloid leukemia
Pathways in cancer
AGE-RAGE signaling pathway in diabetic complications
Legionellosis
Leishmaniasis
Viral myocarditis
Osteoclast differentiation
Toxoplasmosis
Coronavirus disease
Complement and coagulation cascades
Enrichment score
Transcription factors
TP53
IRF8
SOX2
CEBPD
ZBTB7A
KLF4
RUNX1
UBTF
GATA2
SPI1
GATA1
0.000129
3
0.000041
61
0.000028
42
0.000023
86
0.000005
782
0.000003
331
0.000002
708
8.64E-07
4.95E-07
1.08E-07
7.54E-11
p-value significance
GSE44770_downregulated genes
Pathways
Citrate cycle (TCA cycle)
Terpenoid backbone biosynthesis
Synthesis and degradation of ketone bodies
Pathways of neurodegeneration
Huntington disease
Oxidative phosphorylation
Parkinson disease
Nicotine addiction
Proteasome
Retrograde endocannabinoid signaling
Enrichment score
Transcription factors
SMAD4
CREB1
MAX
NRF1
CREB1
TAF1
AR
GABPA
SUZ12
REST
REST
0.002202
0.001196
0.000935
1
0.000865
6
0.000044
18
0.000019
68
0.000009
546
5.62E-07
4.34E-07
1.06E-08
7.25E-11
p-value significance
GSE48350_upregulated genes
Pathways
Phagosome
Chronic myeloid leukemia
Osteoclast differentiation
Hippo signaling pathway
Pathways in cancer
Viral myocarditis
Allograft rejection
Cellular senescence
Inflammatory bowel disease
Leishmaniasis
Enrichment score
Transcription factors
ESR1
AR
TCF3
SALL4
CHD1
SOX2
STAT3
TCF3
SPI1
GATA1
UBTF
0.000025
64
0.000021
07
0.000019
22
0.000016
04
0.000014
24
0.000004
282
8.13E-07
5.45E-07
1.27E-07
2.74E-08
1.17E-17
p-value significane
GSE48350_downregulated genes
Pathways
Phagosome
Chronic myeloid leukemia
Osteoclast differentiation
Hippo signaling pathway
Pathways in cancer
Viral myocarditis
Allograft rejection
Cellular senescence
Inflammatory bowel disease
Leishmaniasis
Enrichment score
Transcription factors
ESR1
AR
TCF3
SALL4
CHD1
SOX2
STAT3
TCF3
SPI1
GATA1
UBTF
0.000025
64
0.000021
07
0.000019
22
0.000016
04
0.000014
24
0.000004
282
8.13E-07
5.45E-07
1.27E-07
2.74E-08
1.17E-17
p-value significane

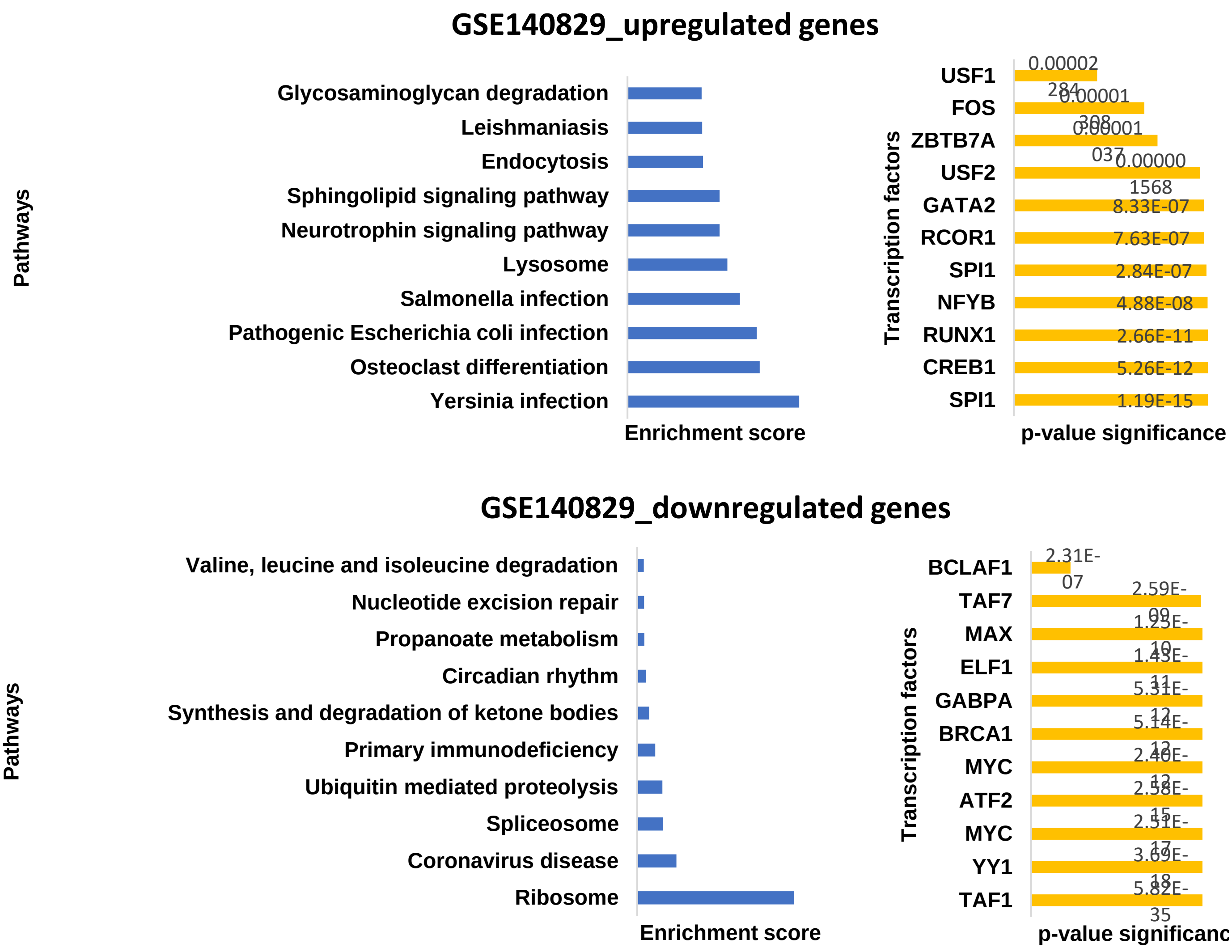

GSE140829_upregulated genes
Pathways
Glycosaminoglycan degradation
Leishmaniasis
Endocytosis
Sphingolipid signaling pathway
Neurotrophin signaling pathway
Lysosome
Salmonella infection
Pathogenic Escherichia coli infection
Osteoclast differentiation
Yersinia infection
Enrichment score
Transcription factors
USF1
FOS
ZBTB7A
USF2
GATA2
RCOR1
SPI1
NFYB
RUNX1
CREB1
SPI1
0.00002284
0.00001308
0.00001037
0.000001568
8.33E-07
7.63E-07
2.84E-07
4.88E-08
2.66E-11
5.26E-12
1.19E-15
p-value significance
GSE140829_downregulated genes
Pathways
Valine, leucine and isoleucine degradation
Nucleotide excision repair
Propanoate metabolism
Circadian rhythm
Synthesis and degradation of ketone bodies
Primary immunodeficiency
Ubiquitin mediated proteolysis
Spliceosome
Coronavirus disease
Ribosome
Enrichment score
Transcription factors
BCLAF1
TAF7
MAX
ELF1
GABPA
BRCA1
MYC
ATF2
MYC
YY1
TAF1
2.31E-07
2.59E-09
1.23E-10
1.43E-11
5.31E-12
5.14E-12
2.40E-12
2.58E-15
2.51E-17
3.69E-18
5.82E-35
p-value significanc
