# Supplementary material for: Integrated multi-omics analysis of Alzheimer’s disease shows molecular signatures associated with disease progression and potential therapeutic targets
Source: Sci Rep. 2023 Mar 6;13:3695. doi: 10.1038/s41598-023-30892-6 (PMC9986671; doi:10.1038/s41598-023-30892-6)

Integrated multi-omics analysis of Alzheimer's disease shows molecular signature associated with disease progression and potential therapeutic targets

Pradeep Kodam[1], Sai Swaroop. R[2], Sai Sanwid Pradhan[2], Venketesh Sivaramakrishnan[2*], Ramakrishna Vadrevu[1*]

[1]Department of Biological Sciences, Birla Institute of Technology and Science Pilani, Hyderabad Campus, Jawahar Nagar, Hyderabad, 500078 Telangana, India

[2]Disease Biology Lab, Department of Biosciences, Sri Sathya Sai Institute of Higher Learning, Prasanthi Nilayam, Anantapur, 515134 Andhra Pradesh, India

[*]Corresponding author

* Venketesh Sivaramakrishnan: s.venketesh@gmail.com

* Ramakrishna Vadrevu: vrk@hyderabad.bits-pilani.ac.in

Keywords: Alzheimer's disease, Integrated multi-omics, Vitamin-cofactor analysis, mice model, neurodegenerative disease.

Supplementary-5 : Kinase and transcription factor enrichment analysis of transcriptomic datasets GSE5281, GSE36980, GSE44770, GSE48350 and GSE140829 respectively.

Kinases and Transcription factors

GSE5281: Brain regions: 1) entorhinal cortex 2) hippocampus 3) medial temporal gyrus 4) posterior cingulate 5) superior frontal gyrus and 6) primary visual cortex.

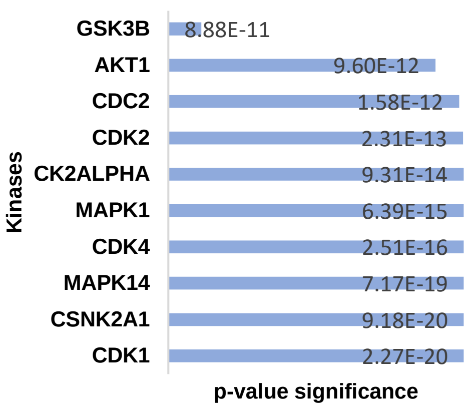

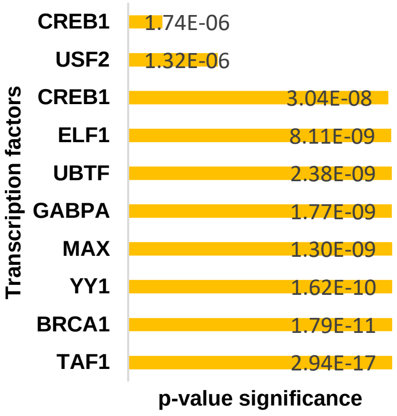

GSE36980: Brain regions : 1)frontal cortex 2) temporal cortex 3).hippocampus

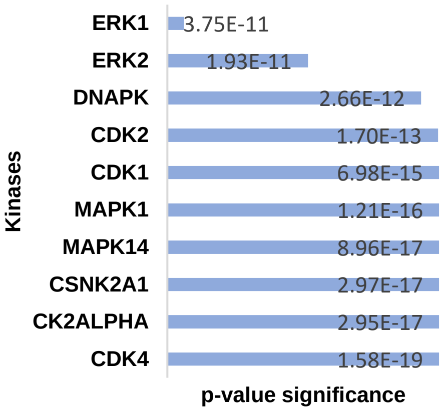

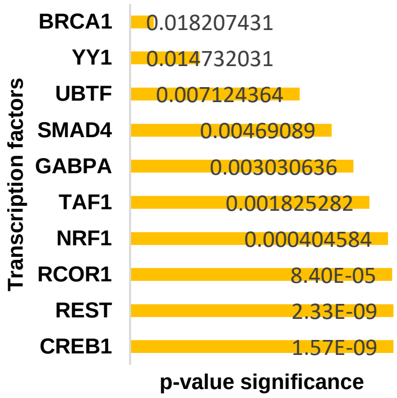

GSE44770: Brain regions: 1)dorsolateral prefrontal cortex 2). visual cortex 3). cerebellum

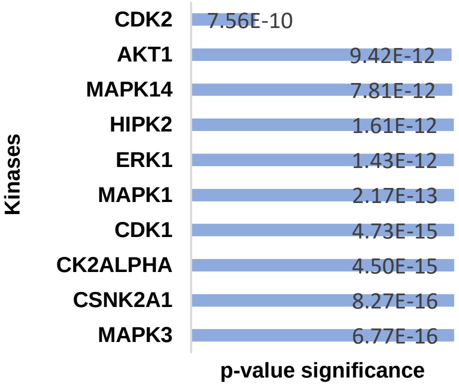

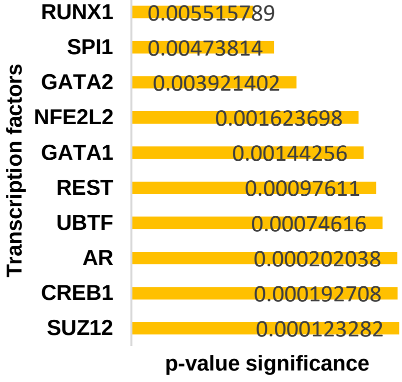

GSE48350: Brain regions: 1)hippocampus 2).entorhinal cortex 3).superior frontal cortex 4).post-central gyrus

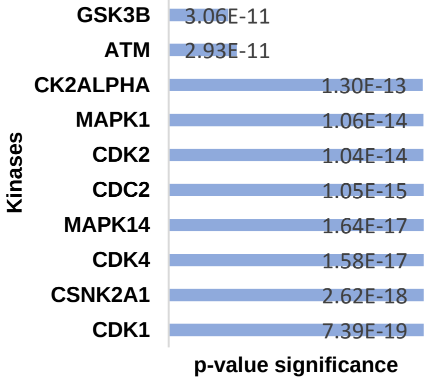

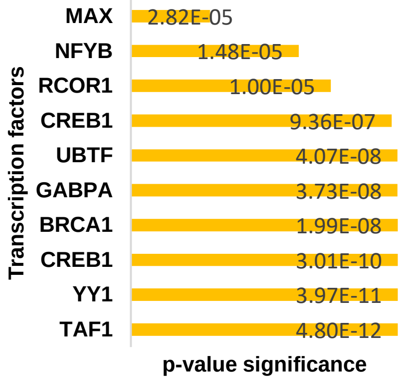

GSE140829: peripheral blood gene expression data

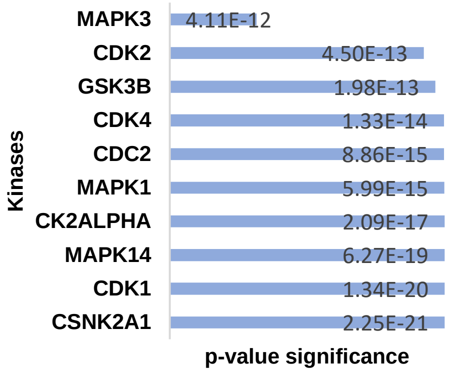

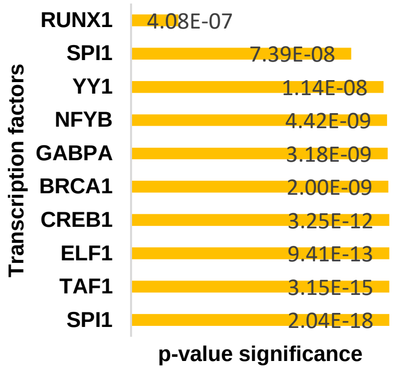

Supplement: Supplementary file 5 — Supplementary Information 5. [file 41598_2023_30892_MOESM5_ESM.pdf]
