# Supplementary material for: Integrated multi-omics analysis of Alzheimer’s disease shows molecular signatures associated with disease progression and potential therapeutic targets
Source: Sci Rep. 2023 Mar 6;13:3695. doi: 10.1038/s41598-023-30892-6 (PMC9986671; doi:10.1038/s41598-023-30892-6)
Supplement: Supplementary file 12 — Supplementary Information 12. [file 41598_2023_30892_MOESM12_ESM.pdf]

Integrated multi-omics analysis of Alzheimer's disease shows molecular signature associated with disease progression and potential therapeutic targets

Pradeep Kodam[1], Sai Swaroop. R[2], Sai Sanwid Pradhan[2], Venketesh Sivaramakrishnan[2*], Ramakrishna Vadrevu[1*]

[1]Department of Biological Sciences, Birla Institute of Technology and Science Pilani, Hyderabad Campus, Jawahar Nagar, Hyderabad, 500078 Telangana, India

[2]Disease Biology Lab, Department of Biosciences, Sri Sathya Sai Institute of Higher Learning, Prasanthi Nilayam, Anantapur, 515134 Andhra Pradesh, India

[*]Corresponding author

* Venketesh Sivaramakrishnan: s.venketesh@gmail.com

* Ramakrishna Vadrevu: vrk@hyderabad.bits-pilani.ac.in

Keywords: Alzheimer's disease, Integrated multi-omics, Vitamin-cofactor analysis, mice model, neurodegenerative disease.

Supplementary-12 : Detailed information of all the datasets analysed in the present study.

Human - Transcriptomics

Dataset	Total number of samples	AD samples	Control samples	Sex<br>AD(Alzheimer's disease)<br>CTRL(Control)		Age<br>AD(Alzheimer's disease)<br>CTRL(Control)				Tissues
GSE5281	161	68	93	Male :<br>50 (AD), 53 (CTRL)	Female :<br>37 (AD), 21 (CTRL)	Female AD :<br>70 - 95 years	Female CTRL :<br>73 - 102 years	Male AD :<br>68 - 97 years	Male CTRL:<br>63 - 85 years	Entorhinal cortex, hippocampus, medial temporal gyrus, posterior cingulate, superior frontal gyrus and primary visual cortex.
GSE36980	80	33	47	Male :<br>15 (AD), 22 (CTRL)	Female :<br>18 (AD), 25 (CTRL)	Female AD :<br>84 – 105 years	Female CTRL :<br>54 – 100 years	Male AD :<br>83 – 99 years	Male CTRL:<br>55 – 89 years	Frontal cortex, Temporal cortex, Hippocampus
GSE44770	230	129	101	Male :<br>62 (AD), 82 (CTRL)	Female :<br>67 (AD), 19 (CTRL)	Female AD :<br>60 - 100 years	Female CTRL :<br>53 - 95 years	Male AD :<br>55 - 98 years	Male CTRL:<br>22 - 106 years	Pre-frontal cortex
GSE48350	253	204	249	Male :<br>33 (AD), 91 (CTRL)	Female :<br>47 (AD), 82 (CTRL)	Female AD :<br>61 -88 years	Female CTRL :<br>62 – 89 years	Male AD :<br>61 – 87 years	Male CTRL:<br>61 – 86 years	Hippocampus, Entorhinal cortex, Superior frontal cortex, Post-central gyrus
GSE140829	587	80	173	Male :<br>100 (AD), 110 (CTRL)	Female :<br>104 (AD), 139 (CTRL)	Female AD :<br>60 – 95 years	Female CTRL :<br>26 – 99 years	Male AD :<br>76 – 94 years	Male CTRL:<br>20 – 97 years	Whole blood

Human - Proteomics

Tissue	Dataset	Total number of samples		AD samples	Control samples
Blood	Proteomics Analysis of Blood Serums from Alzheimer's Disease Patients Using iTRAQ Labeling Technology	60	Number of samples	30	30
			Gender	male : female (3:2)	
			Age	65 - 85 years	
Brain	Deep Multilayer Brain Proteomics Identifies Molecular Networks in Alzheimer's Disease Progression	90	Number of samples	36	54
			Gender	16 male 20 female	30 male 24 female
			Age	60 - 99 years	69 – 98 y
CSF	Integrated proteomics reveals brain-based cerebrospinal fluid biomarkers in asymptomatic and symptomatic Alzheimer's disease	40	Number of samples	20	20
			Gender	12 male 8 female	11 male 9 female
			Age	65.6 ± 11.6 years	69.2 ± 9.0 years

Human - Metabolomics

Tissue	Dataset	Total number of samples		AD samples	Control samples
Blood	Metabolite profiling for the identification of altered metabolic pathways in Alzheimer's disease	44	Number of samples	23	21
			Gender	8 male 15 female	9 male 12 female
			Age	79.52 ± 5.9 years	72.1 ± 5.4 years
Brain	Unbiased metabolomic investigation of alzheimers disease brain points to dysregulation of mitochondrial aspartate metabolism	40	Number of samples	21	19
			Gender	9 male 12 female	12 male 7 female
			Age	82.4 ± 6.7 years	83.4 ± 6.4 years
CSF	Comparative analysis of cerebrospinal fluid metabolites in Alzheimer's disease and idiopathic normal pressure hydrocephalus in a Japanese cohort	58	Number of samples	39	19
			Gender	14 male 25 female	9 male, 10 female
			Age	47 - 86	70 - 86

Mice - Proteomics

Tissue	Dataset	Total number of samples		AD samples	Control samples
Blood	Proteomic Analysis of Serum Proteins in Triple Transgenic Alzheimer’s Disease Mice: Implications for Identifying Biomarkers for Use to Screen Potential Candidate Therapeutic Drugs for Early Alzheimer’s Disease	12	Number of samples	12	4
			Gender	12 male	4 male
			Age	5 months	5 months
			Strain	3xTg-AD mice expressing the human mutations APPSwe, PS1M146V, and TauP301L (strain: B6; 129-Psen1tm1Mpm Tg [APPSwe, tauP301L]	WT mice (strain: B6129SF2/J)
Brain	iTRAQ Analysis of Complex Proteome Alterations in 3xTgAD Alzheimer’s Mice: Understanding the Interface between Physiology and Disease	16	Number of samples	8	8
			Gender	8 male	8 female
			Age	16 month	16 month
			Strain	3xTgAD mice	C57/BL6 control

Mice - Metabolomics

Tissue	Dataset	Total number of samples		AD samples	Control samples
Blood	Alzheimer's disease like pathology has transient effects on the brain and blood metabolome	82	Number of samples	41	41
			Age	6months (9 samples).8,10,12,18 months (8 samples each respectively)	6months (9 samples).8,10,12,18 months (8 samples each respectively)
			Strain	APP/PS1 mice are a transgenic C57BL/6J and coexpressing the Swedish mutation (K595N/M596L) and the deltaE9 PS1 exon deletion	Mice not expressing the transgene were used as WT controls.
Brain	Metabolomic screening of regional brain alterations in the APP/PS1transgenic model of Alzheimer's disease by direct infusion mass spectrometry	60	Number of samples	30	30
			Gender	13 male 17 female	15 male 15 female
			Age	6 months	6 months
			Strain	Transgenic APP/PS1 mice (C57BL/6 background) were generated by expressing the Swedish mutation of APP together with PS1 deleted in exon9	age-matched wild-type mice of the same genetic background (C57BL/6)
CSF	Defects in Mitochondrial Dynamics and Metabolomic Signatures of Evolving Energetic Stress in Mouse Models of Familial Alzheimer's Disease	12	Number of samples	9	3
			Gender	2 male 7 female	1 male 2 female
			Age	16 and 36 months	16 and 36 months
			Strain	Transgenic mice (C57B6) that expressed mutant human APP695 containing a double mutation (K670N, M671L). PS1 mice :mutant human PS1 containing a single mutation (M146L). APP/PS1, were produced in house by crossbreeding of homozygous PS1 and heterozygous APP mice.	Littermates that did not carry transgenes were used as non transgenic control
