# Supplementary material for: Integrated multi-omics analysis of Alzheimer’s disease shows molecular signatures associated with disease progression and potential therapeutic targets
Source: Sci Rep. 2023 Mar 6;13:3695. doi: 10.1038/s41598-023-30892-6 (PMC9986671; doi:10.1038/s41598-023-30892-6)
Supplement: Supplementary file 13 — Supplementary Information 13. [file 41598_2023_30892_MOESM13_ESM.pdf]

Integrated multi-omics analysis of Alzheimer's disease shows molecular signature associated with disease progression and potential therapeutic targets

Pradeep Kodam[1], Sai Swaroop. R[2], Sai Sanwid Pradhan[2], Venketesh Sivaramakrishnan[2*], Ramakrishna Vadrevu[1*]

[1]Department of Biological Sciences, Birla Institute of Technology and Science Pilani, Hyderabad Campus, Jawahar Nagar, Hyderabad, 500078 Telangana, India

[2]Disease Biology Lab, Department of Biosciences, Sri Sathya Sai Institute of Higher Learning, Prasanthi Nilayam, Anantapur, 515134 Andhra Pradesh, India

[*]Corresponding author

* Venketesh Sivaramakrishnan: s.venketesh@gmail.com

* Ramakrishna Vadrevu: vrk@hyderabad.bits-pilani.ac.in

Keywords: Alzheimer's disease, Integrated multi-omics, Vitamin-cofactor analysis, mice model, neurodegenerative disease.

Supplementary-13: Detailed information of all the datasets analyzed in the present study.

Omics	Organism	Tissue	Source	
Transcriptomics	Human	Blood	GSE140829 (peripheral blood gene expression data)	
		Brain	GSE5281	Brain regions: 1) entorhinal cortex 2) hippocampus 3) medial temporal gyrus 4) posterior cingulate 5) superior frontal gyrus and 6) primary visual cortex.
			GSE36980	Brain regions : 1)frontal cortex 2) temporal cortex 3).hippocampus
			GSE44770	Brain regions: 1)dorsolateral prefrontal cortex 2). visual cortex 3). cerebellum
			GSE48350	Brain regions: 1)hippocampus 2).entorhinal cortex 3).superior frontal cortex 4).post-central gyrus
Proteomics	Human	Blood	Proteomics Analysis of Blood Serums from Alzheimer’s Disease Patients Using iTRAQ Labeling Technology	
		Brain	Deep Multilayer Brain Proteomics Identifies Molecular Networks in Alzheimer’s Disease Progression	
		CSF	Integrated proteomics reveals brain-based cerebrospinal fluid biomarkers in asymptomatic and symptomatic Alzheimer’s disease	
	Mice	Blood	Proteomic analysis of serum proteins in triple transgenic alzheimer’s disease mice: Implications for identifying biomarkers for use to screen potential candidate therapeutic drugs for early alzheimer’s disease.	
		Brain	iTRAQ analysis of complex proteome alterations in 3xTgAD Alzheimer’s mice: Understanding the interface between physiology and disease.	
Metabolomics	Human	Blood	Metabolite profiling for the identification of altered metabolic pathways in Alzheimer’s disease	
		Brain	Unbiased Metabolomic Investigation of Alzheimer's Disease Brain Points to Dysregulation of Mitochondrial Aspartate Metabolism	
		CSF	Comparative analysis of cerebrospinal fluid metabolites in Alzheimer’s disease and idiopathic normal pressure hydrocephalus in a Japanese cohort	
	Mice	Blood	Alzheimer’s disease-like pathology has transient effects on the brain and blood metabolome.	
		Brain	Metabolomic screening of regional brain alterations in the APP/PS1 transgenic model of Alzheimer’s disease by direct infusion mass spectrometry.	
		CSF	Defects in mitochondrial dynamics and metabolomic signatures of evolving energetic stress in mouse models of familial alzheimer’s disease.	
