# Supplementary material for: Integrated multi-omics analysis of Alzheimer’s disease shows molecular signatures associated with disease progression and potential therapeutic targets
Source: Sci Rep. 2023 Mar 6;13:3695. doi: 10.1038/s41598-023-30892-6 (PMC9986671; doi:10.1038/s41598-023-30892-6)
Supplement: Supplementary file 14 — Supplementary Information 14. [file 41598_2023_30892_MOESM14_ESM.pdf]

Integrated multi-omics analysis of Alzheimer's disease shows molecular signature associated with disease progression and potential therapeutic targets

Pradeep Kodam[1], Sai Swaroop. R[2], Sai Sanwid Pradhan[2], Venketesh Sivaramakrishnan[2*], Ramakrishna Vadrevu[1*]

[1]Department of Biological Sciences, Birla Institute of Technology and Science Pilani, Hyderabad Campus, Jawahar Nagar, Hyderabad, 500078 Telangana, India

[2]Disease Biology Lab, Department of Biosciences, Sri Sathya Sai Institute of Higher Learning, Prasanthi Nilayam, Anantapur, 515134 Andhra Pradesh, India

[*]Corresponding author

* Venketesh Sivaramakrishnan: s.venketesh@gmail.com

* Ramakrishna Vadrevu: vrk@hyderabad.bits-pilani.ac.in

Keywords: Alzheimer's disease, Integrated multi-omics, Vitamin-cofactor analysis, mice model, neurodegenerative disease.

Supplementary-14 : Overall summary of the multi-omics analysis and top pathways associated with cell types discussed. Reduced levels of TCA metabolites, riboflavin, and Aβ binding to neurons leads to recruitment of kinases such as FYN, that activate microglia, as well as the suppression of transcription factors like CREB and TCF3 all, contribute to synaptic dysfunction. In an astrocyte, L-cysteine interacts with glutamate and glycine to produce GSH, which lowers oxidative stress and enhances cognitive function. Low levels of riboflavin, TCA metabolites, and cysteine in AD brains contribute to cognitive decline. The synthesis of precursors of acetylcholine, a neurotransmitter closely associated with cognitive function, requires both acetyl-CoA and succinyl-CoA. Tau protein is phosphorylated by FYN and GSK3B, which also maintains microtubule integrity. Cognitive impairment is linked to TCF3 dysregulation. The generation of ROS is linked to the metabolism of tryptophan, and oxidative stress is related to the metabolism of pyruvate. CK2 in astrocytes, which is implicated in the neuroinflammatory response, MAPK in microglia, which is responsible for the generation of pro-inflammatory cytokines, and JNK are all activated in response to Aβ. Neuronal cell death was found in AD brains with lower pantothenate levels. MYC expression is elevated, which kills neurons. Memory loss in AD patients is caused by low levels of vitamin B6, pyruvate, tyrosine, and tryptophan. AKT and CREB deregulation can lead to memory loss.

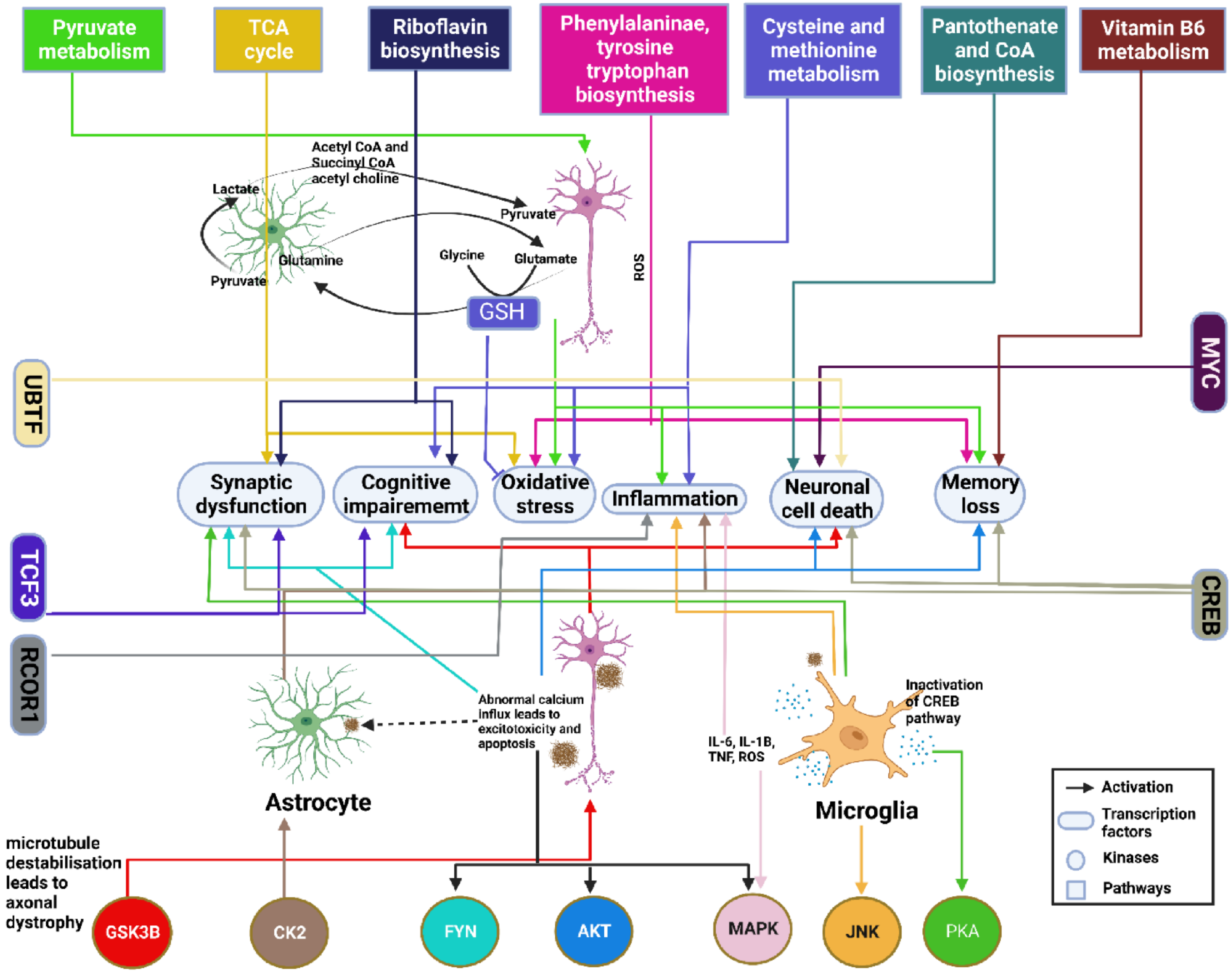

GABAergic neuron

Neuroactive ligand-receptor interaction
Nicotine addiction
Folate biosynthesis
Amphetamine addiction
Glutamatergic synapse

Glutamatergic neuron

Circadian rhythm
Various types of N-glycan biosynthesis
Axon guidance
Cocaine addiction
N-Glycan biosynthesis

Microglia

Riboflavin metabolism
Osteoclast differentiation
Primary immunodeficiency
Complement and coagulation cascades
Platelet activation

Astrocyte

Renin-angiotensin system
Bladder cancer
Signaling pathways regulating pluripotency of stem cells
Phospholipase D signaling pathway
Vasopressin-regulated water reabsorption
